# Biochar Enhances Fischer–Tropsch Electrofuels from CO_2_ and Renewable Energy

**DOI:** 10.1021/acssuschemeng.5c03540

**Published:** 2025-10-23

**Authors:** Marina T. Chagas, Juan D. Medrano-García, Gonzalo Guillén-Gosálbez

**Affiliations:** Institute for Chemical and Bioengineering, Department of Chemistry and Applied Biosciences, 27219 ETH Zurich, Vladimir Prelog Weg 1, Zurich 8093, Switzerland

**Keywords:** Fischer−Tropsch electrofuels, e-fuels, Boudouard reaction, biochar, efficient hydrogen usage, life cycle assessment (LCA), system expansion, techno-economic assessment (TEA)

## Abstract

Electrofuels have emerged as a promising category of alternative fuels for decarbonizing long-distance modes of transport where electrification opportunities might be limited. Despite the favorable environmental performance, their high cost, driven mostly by the expensive electrolytic hydrogen (H_2_), still poses a challenge to their widespread adoption. Here, we propose a novel approach based on carbon dioxide (CO_2_) gasification of biochar via the reverse Boudouard reaction to decrease the H_2_ demand in Fischer–Tropsch (FT) electrofuel synthesis. We adopt a system expansion approach and assess the life-cycle environmental impacts and techno-economic feasibility of this route considering the replacement of different end-uses of biochar. The comparison to the standard reverse water–gas shift (RWGS) configuration showcases that shifting to the Boudouard route could lead to a reduction in cost, carbon footprint, and impact on human health and ecosystems quality. Nevertheless, collateral damage toward resource depletion could take place depending on the choice of the expanded system for the analysis. In our best case scenario, we improve the global warming impact by 11% and lower the cost by 10% while achieving damage reductions in the range of 10–17% to human health, ecosystem quality, and resource scarcity. Overall, this work sheds light on the potential economic and environmental benefits of a more efficient material integration among processes. Moreover, our results hint at the importance of defining proper system expansion scenarios in assessing alternative technologies, as varying system boundaries could yield different assessment outcomes.

## Graphical abstract

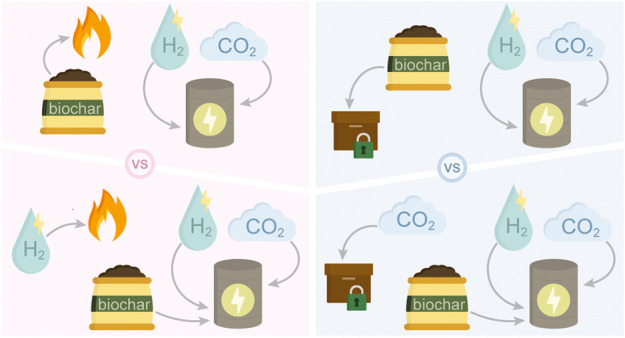

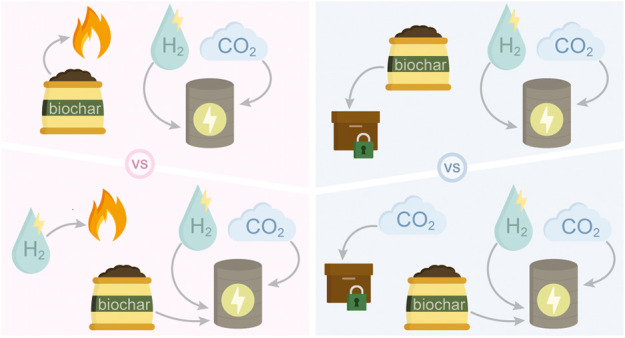

## Introduction

Transport heavily relies on fossil fuels and accounts for more than a third of the CO_2_ emissions from end-use sectors.^1^ In 2022, over 95% of the energy consumption in the sector stemmed from fossil fuels.^2^ Reaching net-zero emissions by 2050 in accordance with the Paris Agreement requires transportation sector emissions to fall by about 25% by 2030.^1^ Ongoing decarbonization efforts include scaling up the use of low-emissions synthetic fuels, especially for long-distance modes of transport such as shipping and aviation, which are hard to abate and where the demand for high-energy-density liquid fuels is expected to remain strong.^3,4^

A key category of synthetic fuels is electrofuels, also known as e-fuels, which are produced from carbon dioxide (CO_2_) as the main carbon source and hydrogen (H_2_) derived from water electrolysis. As drop-in replacements for conventional fossil fuels, they can make use of existing infrastructure and engines, thus reducing the cost of decarbonization.^3,5,6^ For electrofuels to reduce carbon emissions relative to their fossil analogs, they need to rely on renewable carbon sources, such as biogenic CO_2_ or, more commonly, CO_2_ captured directly from the air,^3,5^ where the latter requires H_2_ obtained via electrolysis powered by renewable low- or zero-carbon energy sources.

One of the most established routes for producing liquid electrofuels is the Fischer–Tropsch (FT) synthesis, in which synthesis gas or syngas—a mixture of carbon monoxide (CO) and H_2_—is converted into a mix of linear and branched hydrocarbons and oxygenated products.^4,7^ To this end, an initial step is required to produce the syngas. In the case of electrofuels, this is typically achieved through the reverse water–gas shift (RWGS) reaction, in which CO_2_ is converted to CO using H_2_.^8^

Previous studies indicate that the production cost of electrolytic H_2_ is the primary driver of the total cost of green chemicals, such as methanol,^9–11^ ammonia,^12^ and ethylene,^13^ as well as green fuels.^14–16^ The capital costs of the electrolyzer and the electricity demand of electrolysis make electrolytic H_2_ expensive, thereby compromising the economic viability of these chemicals and fuels.^14,17^

Atsonios et al.^9^ found that electrolyzer costs account for 20% of the total cost of methanol, which is approximately 2.5 times more expensive than its fossil-based counterpart. Similarly, D’Angelo et al.^12^ estimated that green H_2_ constitutes 68–92% of green ammonia production costs, leading to a price increase of 42–891% compared to the fossil alternative. González-Garay et al.^10^ reported a share of up to 73% for H_2_ in the total green methanol production cost, which was up to 2.6-fold higher than the fossil-based analog depending on the electricity source used for electrolysis.

Likewise, König et al.^14^ investigated the production of liquid hydrocarbons from CO_2_ and wind-powered water electrolysis, finding that production costs could be up to seven times higher than the current market price, with the electrolyzer and wind power as the main cost drivers. Medrano-García et al.^15^ found that the electricity requirements combined with the electrolyzer capital costs account for 74–84% of the total expenses for electrodiesel production from captured CO_2_, making it at least five times more expensive than the fossil alternative. Finally, Freire Ordóñez et al.^16^ reported FT e-jet fuels with at least 5.4-fold the cost of the fossil counterpart, mainly due to the high investment cost of green H_2_. For this reason, while the RWGS reaction provides an established route to reduce CO_2_ to CO and thus obtain syngas, its additional H_2_ requirements might limit the economic feasibility of FT electrofuels against fossil fuels.

Electrochemical CO_2_ reduction, also known as CO_2_ electrolysis, is emerging as a promising alternative for the synthesis of energy carriers such as CO, formic acid, and methanol from CO_2_.^18,19^ In this context, the production of syngas by co-electrolysis of water and CO_2_ using solid oxide electrolysis cells has been gaining attention.^20^ One of the major challenges is controlling the composition of the syngas, as the main electrolysis reactions and side reactions occur simultaneously and their contribution depends on different factors such as cell materials and structure, and operating conditions.^20,21^ Moreover, as both CO_2_ and water electrolysis processes present similar energy requirements, replacing the RWGS reaction with CO_2_ electrolysis in the context of FT electrofuel production would reduce the H_2_ requirements, but the overall energy input would remain approximately the same.^22,23^

In this study, we propose an alternative route, the reverse Boudouard reaction, for the CO production step.^24^ In essence, CO_2_ reacts directly with solid carbon to yield CO, thus reducing the reliance on H_2_ in the overall synthesis framework. In addition to potential cost savings, as will be discussed later in this article, this reaction route could improve the environmental performance of the electrofuels depending on the selected solid carbon source. Here, we propose the use of biochar, a carbonaceous solid material obtained by heating biomass to a temperature over 350 °C under conditions of controlled and limited oxidant concentrations to prevent combustion.^25^ These processes can be classified as either pyrolysis, when there are no oxidants present and biochar can be obtained as the main product, or gasification, when the oxidant concentrations are sufficient to generate syngas and biochar is thus obtained as a byproduct.^25^

Current applications for biochar include soil enhancement and carbon sequestration, adsorption for water treatment, anaerobic digestion, tar removal, catalysis, and electrochemistry, as well as for energy purposes.^26–28^ Recent studies have investigated the use of biochar for CO_2_ conversion into CO via the reverse Boudouard reaction for the subsequent production of chemicals.^23,24,29^ More specifically, Medrano-García et al.^23^ proposed the production of green methanol based on the standard CO_2_ hydrogenation process integrated with the reverse Boudouard reaction using biochar. To this end, an expanded system assuming the simultaneous production of methanol, biogenic H_2_, and industrial high-temperature heating was considered. As a result, an environmental and economic win–win scenario was reported in comparison to the base green methanol case.^23^ Nevertheless, to the best of the authors’ knowledge, there have been no studies so far that consider the integration of the reverse Boudouard reaction for CO generation for the production of liquid fuels. The novelty of this work, therefore, lies in incorporating the reverse Boudouard reaction into the production of electrofuels via FT synthesis. Furthermore, as the system boundaries can influence the analysis outcomes, we argue that different expanded systems should be studied for a more comprehensive evaluation of technologies and their trade-offs.

In this work, we aim to evaluate the potential of integrating the reverse Boudouard reaction with the FT synthesis for electrofuel production in improving their economic and environmental performance. We carry out a complete economic and environmental assessment of FT electrofuel production from captured CO_2_ and wind-based electrolytic H_2_ employing the reverse Boudouard reaction and compare it to the established route via the RWGS reaction. We adopt a system expansion approach to account for the fuel synthesis, the production of biochar via biomass gasification, and the alternative uses and potential substitutes if this solid carbon source is allocated to fuel synthesis instead of an alternative application. We find that the Boudouard configuration has the potential to reduce both the carbon footprint and the production costs of the electrofuels. Nevertheless, a burden shift to other impact categories may occur depending on the expanded system considered for the analysis.

## Methods

We evaluated the economic and environmental performance of FT electrofuel synthesis considering two pathways (RWGS and Boudouard scenarios) to produce CO from captured CO_2_. To compare the scenarios, we adopted a system expansion approach to consider the alternative use of biochar in the analysis. We developed detailed process simulations with rigorous kinetic models in Aspen HYSYS v11 coupled with MATLAB R2022a and performed heat integration in Aspen Energy Analyzer v11 through pinch analysis. Based on the mass and energy balance results, we modeled the life cycle inventories (LCIs) and carried out a life cycle assessment (LCA) in SimaPro v9.5 using the Ecoinvent v3.5 database and a standard economic assessment considering both capital and operational expenditures (CAPEX and OPEX, respectively). Finally, we performed an uncertainty analysis to assess the impact of uncertain parameters on the economic and environmental assessments results. The following subsections describe the above-mentioned methods.

### Case Studies

We considered two scenarios that differ in the reaction routes for generating syngas for the FT synthesis, namely, the RWGS and the Boudouard scenarios. In the first case, captured CO_2_ and electrolytic H_2_ react via the RWGS reaction (eq 1) for the direct generation of syngas, whose composition can be adjusted afterward with additional H_2_. In the second case, captured CO_2_ reacts first with biochar via the reverse Boudouard reaction (eq 2) to produce CO, which is then mixed with electrolytic H_2_ for the Fischer–Tropsch synthesis.

$${\mathrm{CO}}_{2}+H_{2}⇌\mathrm{CO}+H_{2}O$$ 1

$$C+{\mathrm{CO}}_{2}⇌2\mathrm{CO}$$ 2

As biochar is only required for the electrofuel production in the Boudouard configuration, its other use in the case of the RWGS route should be considered to ensure a fair comparison of scenarios. Additionally, its production should also be taken into account in an expanded system approach. We assumed the biochar to be obtained as a byproduct in biomass gasification for H_2_ production with carbon capture and storage (CCS) and considered two alternative uses for biochar for the system expansion: in high-temperature industrial heating (IH) and as a carbon dioxide removal (CDR) technology. In essence, when biochar is used in the production of electrofuels via the Boudouard route and is therefore not available for other applications, an alternative for industrial heat generation and CDR technology should be proposed.

For the system expansion considering industrial heat generation (Figure 1a), biochar is combusted for heat generation in the scenario in which the RWGS route is selected (RWGS_IH). On the other hand, when Boudouard is the route of choice (B_IH), IH is assumed to be obtained through the combustion of electrolytic H_2_. For the second expanded system (Figure 1b), biochar carbon removal (BCR) is considered the CDR technology when the RWGS reaction is selected (RWGS_CDR), whereas direct air carbon capture and storage (DACCS) is implemented as the CDR option in the scenario employing the Boudouard route (B_CDR).

**Figure 1:**
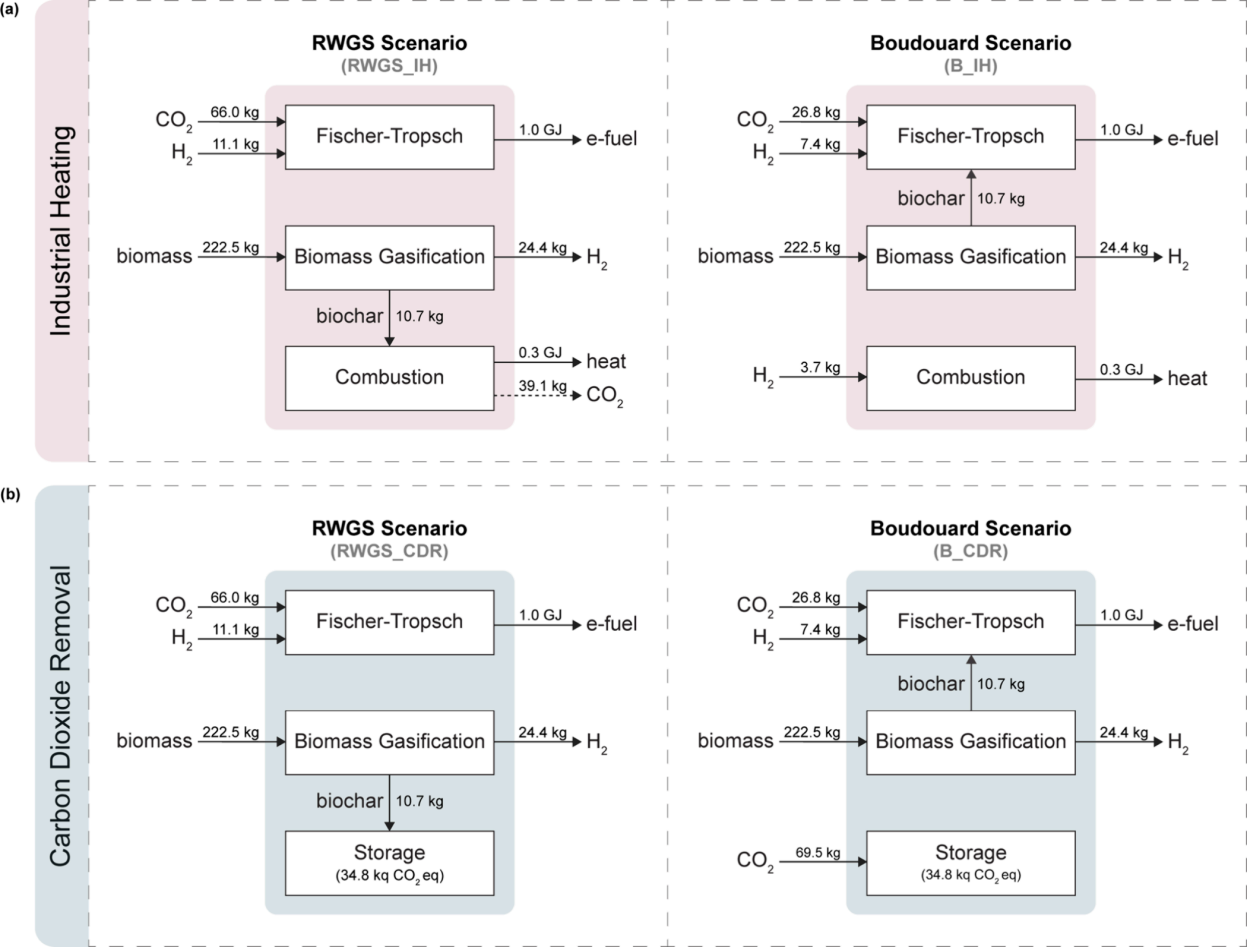
Graphic representation of the four scenarios included in this study. Each scenario includes the production of electrofuels via the RWGS or reverse Boudouard reaction coupled with the FT synthesis, the production of biochar via biomass gasification, and the alternative biochar application or its replacement. The latter defines the two expanded systems: (a) IH and (b) CDR, each of which comprises two scenarios (RWGS and Boudouard).

In total, four scenarios are evaluated in this study, each one of which comprising FT electrofuel production, biochar production, and alternative biochar application or its replacement. The scenarios were evaluated in pairs within the respective expanded systems. Figure 1 provides a visual representation of the expanded systems and scenarios considered.

### Process Modeling

The production of FT electrofuels was simulated in Aspen HYSYS v11 coupled with MATLAB R2022a to enable the use of rigorous kinetic models for the FT reaction system. The Peng–Robinson method was selected for the estimation of the thermodynamic properties.

In general, the production of FT electrofuels can be divided into five sections: syngas production, syncrude production from syngas, wax hydrocracking, separation, and light components combustion. The two process flowsheets considered in this study differ from one another in terms of the first section, where CO is obtained from captured CO_2_. In the RWGS configuration (Figure 2a), CO_2_ from direct air capture (DAC) and H_2_ from wind-based water electrolysis react via the RWGS reaction for the direct generation of syngas. Before entering the RWGS reactor (R-RWGS) in a H_2_/CO_2_ ratio of 4.35,^15^ the pressure of the raw materials streams is adjusted either through a series of three compression stages with intercooling to 40 °C in the case of CO_2_, as it is assumed to be fed at 25 °C and 1 bar,^15^ or through expansion in the case of H_2_, assumed to be fed at 80 °C and 30 bar.^30^ The two feed streams are then mixed and heated to the temperature of R-RWGS, which is modeled as a Gibbs reactor operating at 900 °C and 25 bar.^16^ The conversion per pass in R-RWGS is 84%. After the reactor, the outlet stream is cooled, and the aqueous phase, considered to be wastewater, is separated from the syngas in a flash unit. Extra H_2_ is added to adjust the H_2_/CO molar ratio in the syngas to 2.05.^14^

**Figure 2:**
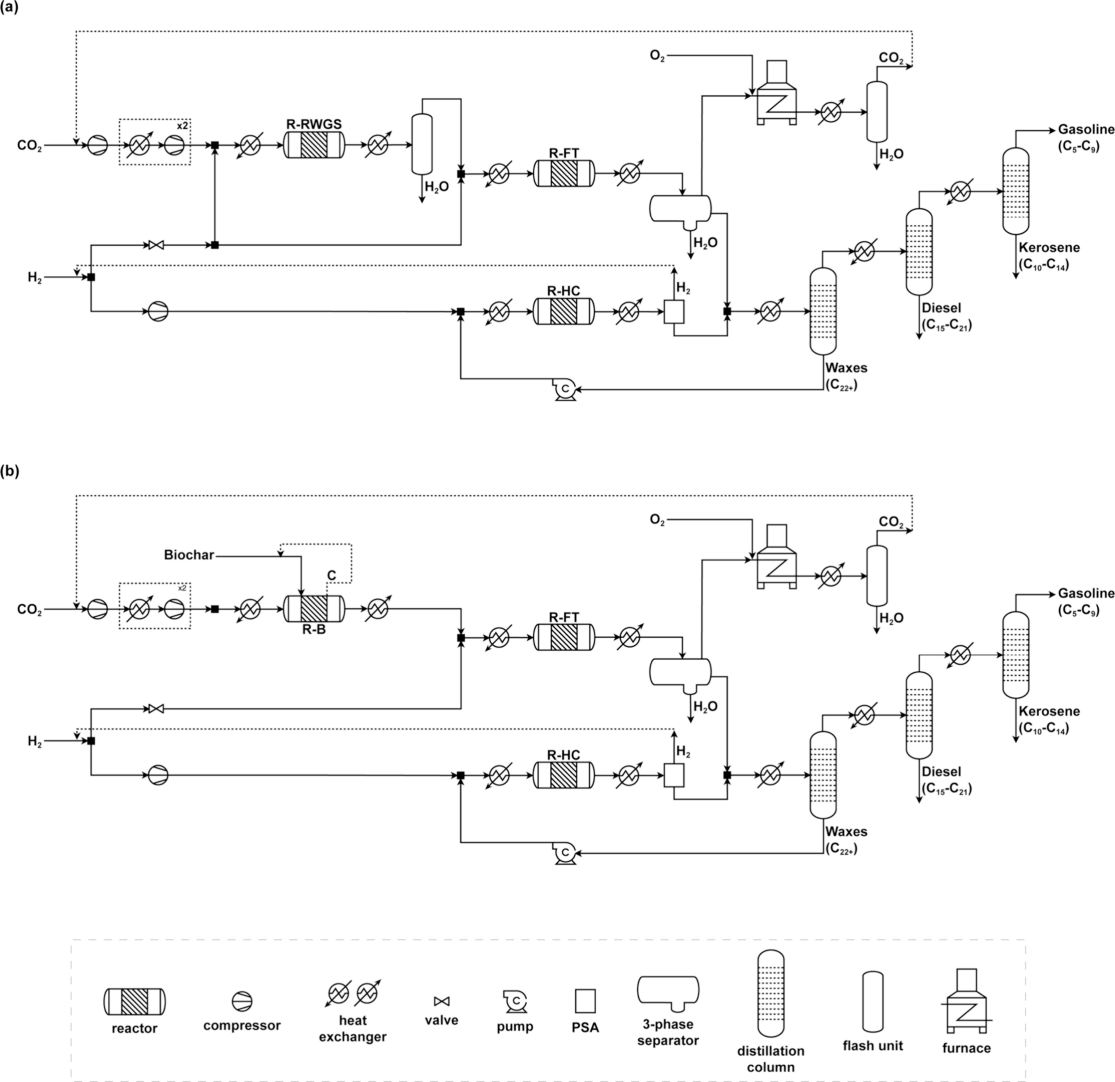
Process flowsheet of FT electrofuel production from captured CO_2_ and electrolytic H_2_. (a) RWGS scenario. (b) Boudouard scenario.

In the case of the Boudouard configuration (Figure 2b), CO_2_ from DAC and biochar, assumed to be pure carbon, react via the reverse Boudouard reaction to produce CO, which is then mixed with H_2_ from wind-based water electrolysis for the fuel synthesis. The CO_2_ feed stream goes through three compression stages with intercooling to 40 °C and is then heated to the reaction temperature (956 °C) and fed to the Boudouard reactor (R-B) along with the biochar in a CO_2_/biochar molar ratio of 1:1. The reactor is modeled as an equilibrium reactor based on Hunt et al.^31^ and operates isothermally at the same pressure as the subsequent FT reactor (25 bar^16^). We model the R-B with a per-pass conversion of 84% to match the CO production rate in R-RWGS, thus ensuring a fair comparison of both systems. Assuming the use of a fixed or fluidized bed reactor, we recycle the unreacted biochar that exits the reactor in the outlet stream. The outlet gas stream, containing CO and unreacted CO_2_, is mixed with a H_2_ feed stream, whose pressure was previously adjusted so that syngas with a H_2_/CO molar ratio of 2.05 ^14^ is obtained.

From this point onward, the description of the process is valid for both process configurations. The syngas is converted in the FT reactor (R-FT) to a range of hydrocarbons, namely, light components (C_1_–C_4_), gasoline (C_5_–C_9_), kerosene (C_10_–C_14_), diesel (C_15_–C_21_), and waxes (C_22+_). We consider a low-temperature FT process, with the reactor operating at 225 °C.^14,16^ The R-FT was modeled in MATLAB based on the kinetic model by Hillestad^32^ and solved considering the formation of 1000 *n*-paraffins and 1000 α-olefins. The *n*-paraffins with the carbon numbers C_31_–C_1000_ and the α-olefins between C_22_ and C_1001_ were lumped into C_30_ and C_21_, respectively. The results of the outlet stream were then exported to Aspen HYSYS. A three-phase separator is used to remove the gaseous and aqueous phases from the main product stream containing the hydrocarbon fractions.

The wax fraction is separated and sent to an upgrading section, where it undergoes hydrocracking to enhance the production of lighter and more valuable hydrocarbons. The hydrocracking reactor (R-HC), operating at 350 °C and 40 bar,^15^ was modeled in MATLAB based on the kinetic model by Mohanty et al.^33^ and Bhutani et al.^34^ With the lumping of α-olefins, the wax fraction is assumed to be composed only of *n*-paraffins, and therefore, only the hydrocracking of *n*-paraffins is considered. Along with the waxes, electrolytic H_2_ is also fed to the reactor according to a H_2_/wax molar ratio of 2.6.^35^ Unreacted H_2_ is separated from the other gaseous components in the outlet stream in a pressure swing adsorption (PSA) system with 99% recovery and 100% purity,^36^ while remaining waxes and lighter hydrocarbon fractions join the main product stream of R-FT and enter the separation section. This is modeled as a series of reboiled absorbers coupled with partial condensers and flash units, where the hydrocarbon fractions are separated following an indirect sequence, with waxes being taken as the bottom product in the first column and sent to the R-HC inlet. The operating conditions were adjusted so that the product distribution was as close as possible to the desired fuel fractions.

The gaseous phase separated from the FT outlet stream is sent to a furnace, where it undergoes oxy-fuel combustion under stoichiometric conditions using part of the oxygen byproduct from the electrolysis process to produce H_2_. Water is removed from the flue gas stream via condensation and taken as wastewater, whereas the CO_2_ is recycled at the beginning of the process for the production of syngas.

Heat integration was performed for each of the process configurations for FT electrofuel production in Aspen Energy Analyzer v11 through pinch analysis.

As previously mentioned, biochar is assumed to be obtained as a byproduct in biomass gasification for H_2_ production with CCS. Simulation results for this process are based on the work by Medrano-García et al.^23^ Likewise, the simulation results for industrial heat generation for the first expanded system considered in this study were also obtained from Medrano-García et al.^23^ for both biochar and H_2_ combustion.

On the other hand, the DACCS process for the second expanded system was simulated in Aspen Plus v12 using the Peng–Robinson equation of state with Boston-Mathias modifications (PR-BM) to estimate the thermodynamic properties. CO_2_ from DAC, originally at 25 °C and 1 bar,^15^ is compressed to 110 bar^23^ in four stages with intercooling to 40 °C. The feed of CO_2_ for storage was calculated to yield the same climate change mitigation potential as the BCR when considering biochar to be pure carbon and to have a stable carbon fraction of 0.89.^25^ A more detailed description of the BCR and DACCS carbon abatement potential calculation can be found in Section A of the Supporting Information.

### Environmental Assessment

We carried out a standard life cycle assessment for the four scenarios in SimaPro v9.5 using Ecoinvent v3.5 database and following the ISO 14040/44 framework.^15^ The main goal was to assess, for each expanded system, the global warming impact (GWI) reduction potential of the Boudouard route for the production of FT electrofuels in comparison with the RWGS route. Additionally, we investigated whether burden-shifting occurs across the endpoint categories (damage to human health, ecosystems quality, and resource depletion) when selecting the reverse Boudouard reaction instead of the RWGS reaction. The assessment methods ReCiPe 2016 v1.03 Midpoint (H) and ReCiPe 2016 v1.03 Endpoint (H) were used, considering a cradle-to-gate scope following a cut-off attributional approach. The ReCiPe is a widely applied life cycle impact assessment (LCIA) methodology that includes characterization factors to quantify environmental impacts via 18 midpoint indicators grouped into three endpoint categories.^37^ The midpoint indicators capture specific environmental issues such as climate change and water use, whereas the three endpoint categories cover damages to human health, ecosystems quality, and resource scarcity.^38^ In this work, we used the hierarchist perspective (100-year time horizon) of the ReCiPe method, which is the most commonly used and is based on scientific consensus regarding environmental impact modeling.^37,38^ We define a functional unit of 1 GJ of electrofuel, 24.4 kg of biogenic H_2_ and 287.3 MJ of high-temperature heat for the IH expanded system, and 1 GJ of electrofuel, 24.4 kg of biogenic H_2_, and 34.8 kg of stored CO_2_-eq for the CDR expanded system.

We computed the LCI for each scenario considering all the inputs and outputs of raw materials, energy, emissions, and waste related to the electrofuel synthesis, biomass gasification, and IH generation or CDR technology. The foreground system was modeled with the mass and energy balance results from the process simulations and complemented with literature sources for the CO_2_ and H_2_ feedstocks, whereas the background system was modeled with the activities from Ecoinvent v3.5. The output data, namely, mass and energy balance results, were exported from the simulations in Aspen HYSYS and Aspen Plus to create the LCIs, which were then imported into SimaPro for the analysis.

Finally, we performed an uncertainty analysis of the four scenarios with a Monte Carlo sampling entailing 2000 different samples generated using the default distributions in SimaPro v9.5 based on the pedigree matrix in Ecoinvent. For each sample, the impact difference between the RWGS and the Boudouard scenarios was calculated, where a positive difference value indicated that there was an impact reduction. The probability of impact reduction was then obtained from the number of samples with a positive difference.

### Economic Assessment

We carried out a techno-economic assessment for the four scenarios by taking into account, for each one, the CAPEX and the OPEX for the electrofuel synthesis, the biomass gasification, and the IH generation or CDR technology. The total annual cost was calculated following the approach described in Sinnott and Towler.^39^ An annual operation of 8000 h was considered to estimate the cost per functional unit.

The CAPEX was estimated from the purchased equipment costs for the main units in each process using standard practices.^39^ The reactor units and the PSA system cost functions were taken from König et al.,^14^ Onel et al.,^40^ and Medrano-García et al.^15^ A plant lifetime of 30 years and an interest rate of 10% were considered in the calculations, and the results were adjusted with the Chemical Engineering Plant Cost Index (CEPCI) to USD 2023.

The OPEX was estimated from the variable and the fixed operational costs, thus accounting for the raw materials, utilities, and other expenses related to labor, maintenance, taxes, insurance, land, and plant overheads.^39^ Purchase prices for the raw materials and utilities were taken from the literature,^41–43^ and the fixed production costs were derived from the CAPEX following the methodology by Sinnott and Towler.^39^

We also performed an uncertainty assessment of the four scenarios to evaluate the impact of key uncertainties on the final cost. To this end, we computed 2000 Monte Carlo simulations by varying the costs of wind-based H_2_ (6.880–8.480 USD/kg), CO_2_ from DAC (0.362–0.623 USD/kg), and the stable carbon fraction of biochar (0.89–1.00) according to the ranges found in the literature.^25,41^ We assumed a uniform distribution for each parameter due to the lack of probability data in the literature. Similarly to the Monte Carlo analysis for the environmental results, the cost difference between the RWGS and the Boudouard scenarios was calculated for each run, with a positive difference value indicating that there was a cost reduction. The probability of cost reduction was then obtained from the number of simulations with a positive difference. More details on the CAPEX, OPEX, and uncertainty analysis calculations can be found in Section C of the Supporting Information.

## Results and Discussion

The results are presented and discussed in two subsections. First, we discuss the environmental assessment results of the RWGS and Boudouard configurations for FT electrofuel production within each expanded system, including FT electrofuel synthesis, biomass gasification, and biochar utilization or its replacement in both IH and CDR. These results are complemented by their respective uncertainty analyses. Then, we discuss the cost breakdown of each scenario and assess the impact of key uncertainties on the results. The simulation results can be found in Section B of the Supporting Information.

### Environmental Assessment Results

This subsection focuses on GWI and the damage assessment impacts of the ReCiPe 2016 methodology. As can be seen in Figure 3, the Boudouard scenario presents the lowest carbon footprint for both expanded systems when compared with the standard RWGS configuration. This reduction stems from the more efficient use of the available resources, that is, H_2_ and biochar, which also translates into an overall decrease in cooling utilities and electricity requirements.

**Figure 3:**
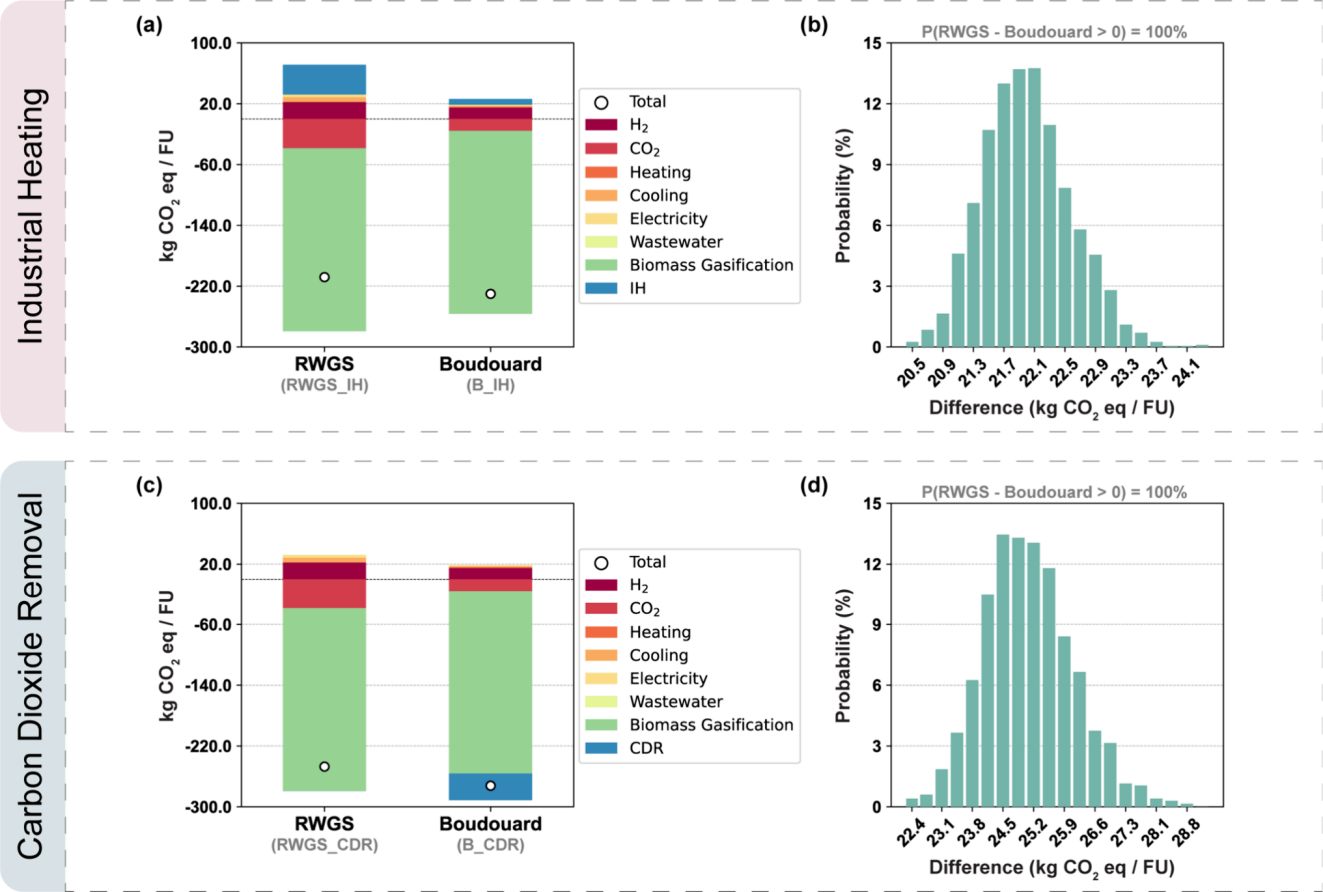
Carbon footprint and uncertainty analysis results per functional unit (FU) for the RWGS and Boudouard scenarios for (a, b) the IH expanded system and (c, d) the CDR expanded system.

Shifting from RWGS to Boudouard implies a reduction in the use of electrolytic H_2_ and DAC CO_2_ in the FT electrofuel synthesis process. The lower H_2_ demand, as previously discussed, is a consequence of it no longer being required for the reduction of CO_2_ to CO, as shown in eq 1. On the other hand, the reduced DAC CO_2_ requirement is a result of biochar also contributing as a carbon source in the process, as can be seen in eq 2. Reducing the demand for DAC CO_2_ also decreases the associated abatement contribution to the global warming impact. However, factors in each expanded system, such as the lower impact of IH via H_2_ combustion and of DACCS as a CDR technology—both compared to their biochar counterparts—help counterbalance this reduction in the abatement contribution, as explained next.

The lower volumes of H_2_ and DAC CO_2_ reduce the power input required by the compressors as well as the cooling required between compression stages. Nevertheless, the shift to the Boudouard configuration introduces a new high-temperature reaction (900 °C RWGS vs 956 °C reverse Boudouard) that increases the heating requirements of the system.

All in all, the carbon footprint reduction when shifting from the RWGS route to the Boudouard route is slightly larger in IH (11%) than in the CDR expanded system (10%). In the case of IH (Figure 3a), the reduction in carbon footprint when comparing the scenarios B_IH and RWGS_IH is mainly due to the lower impact of IH via H_2_ combustion as opposed to biochar combustion. In essence, unlike biochar combustion for heat generation, there are no direct CO_2_ emissions when H_2_ is combusted, which offsets the reduced DAC CO_2_ contribution in terms of the carbon footprint.

On the other hand, in the CDR expanded system (Figure 3c), the lower impact of the CDR technology is the main contributor to the GWI reduction. More specifically, in the B_CDR scenario, there is an additional contribution from DACCS to decrease the GWI, which is added to the impact of biochar production considered in both scenarios because of the expanded system approach.

The lower electrolytic H_2_ demand also plays a role in decreasing the carbon footprint of B_CDR in comparison to that of RWGS_CDR. It is worth noting, however, that this is not the case in the IH scenarios, as the total H_2_ input within the expanded system ends up being virtually the same. This is because the IH generation via H_2_ combustion requires approximately the same amount of H_2_ that is saved in the FT synthesis process when shifting from the RWGS reaction to the reverse Boudouard reaction for CO production.

Figure 3 also shows the uncertainty assessment results regarding GWI for the IH scenarios (Figure 3b) and the CDR scenarios (Figure 3d). For both expanded systems, the Boudouard scenario presents a lower carbon footprint than the RWGS scenario in all of the 2000 sampled backgrounds randomly generated. Thus, it is possible to conclude that regardless of the system expansion considered, the shift from RWGS to Boudouard reduces the climate change impact of the system.

The results of the damage assessment impacts can be found in Figure 4. For the IH expanded system, the Boudouard scenario performs better than the RWGS scenario in all three endpoints, namely, damage to human health, ecosystems quality, and resource scarcity, as depicted in Figure 4a. The improvements are, respectively, 17%, 10%, and 13%. Overall, the impact decrease can be attributed to the use of biochar to convert captured CO_2_ into CO instead of employing it for heat generation, also implying, as aforementioned, lower cooling utilities and electricity requirements for the FT synthesis. Regarding resource depletion, the reduction of DAC CO_2_ input also favors a lower impact. This is because of the heating required for the regeneration of the adsorbent in the DAC process, which is assumed to be provided by natural gas combustion.

**Figure 4:**
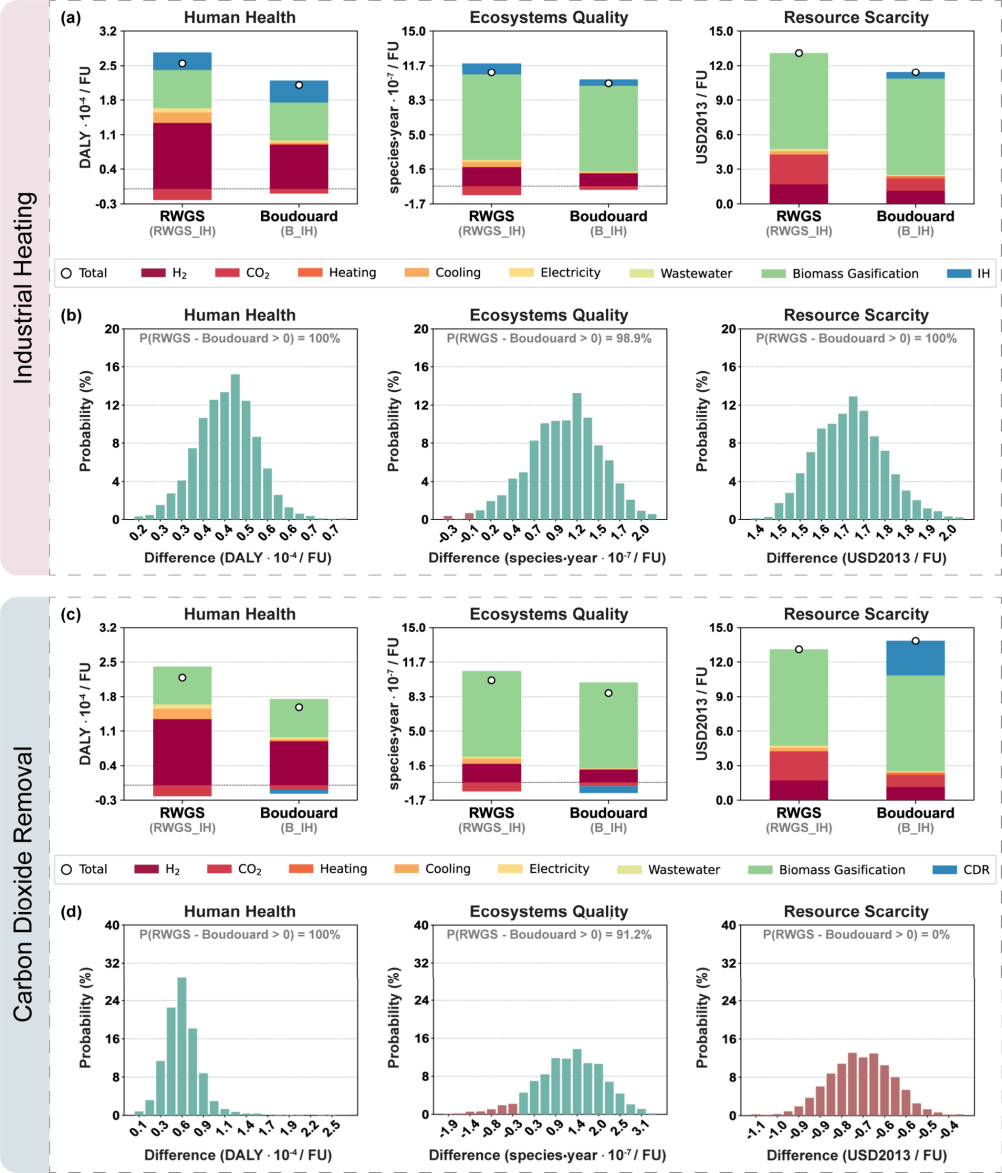
Damage assessment and uncertainty analysis results per functional unit (FU) for RWGS and Boudouard scenarios for (a, b) the IH expanded system and (c, d) the CDR expanded system.

Furthermore, the uncertainty assessment results in Figure 4b show that there is virtually no probability of burden shifting. For all of the 2000 sampled backgrounds randomly generated, the damages to human health and resource depletion were lower when replacing the RWGS with the Boudouard route. Regarding ecosystems quality, the Boudouard scenario outperforms the RWGS scenario in 98.9% of the sampled backgrounds, deeming the potential collateral damage also statistically negligible.

Regarding the system expansion with CDR, Figure 4c shows that when the Boudouard route is used instead of the RWGS route (and, consequently, BCR is replaced by DACCS as the CDR technology) there is a reduction of the damage to human health (28%) and to ecosystems quality (12%) at the cost of burden shifting toward resource scarcity, which increases by 6%. This collateral damage can be attributed to the heating from natural gas required in the CO_2_ desorption step in the DAC process, as previously mentioned.

The uncertainty analysis results in Figure 4d confirm that there is a high likelihood of impact reduction on human health (100%) and ecosystems quality (91.2%) when shifting from the RWGS route with BCR to the Boudouard route with DACCS. Nevertheless, the uncertainty analysis indicates that there is a 100% probability of burden shifting toward resource depletion.

More details on the LCA results can be found in Section D of the Supporting Information.

### Economic Assessment Results

The results of the techno-economic assessment are displayed in Figure 5. Overall, the Boudouard scenario outperforms once again the RWGS independently of the expanded system of choice. As can be seen from the cost breakdown (Figure 5a,c), this reduction is more significant between the IH scenarios (10%) than in the CDR scenarios (6%) due to the high cost of DACCS assumed to replace biochar in a potential CDR usage. That is because of the amount of captured CO_2_ necessary to provide the same abatement potential as BCR (34.8 kg of CO_2_-eq per functional unit) when biochar is used instead as a feedstock for fuel synthesis.

**Figure 5:**
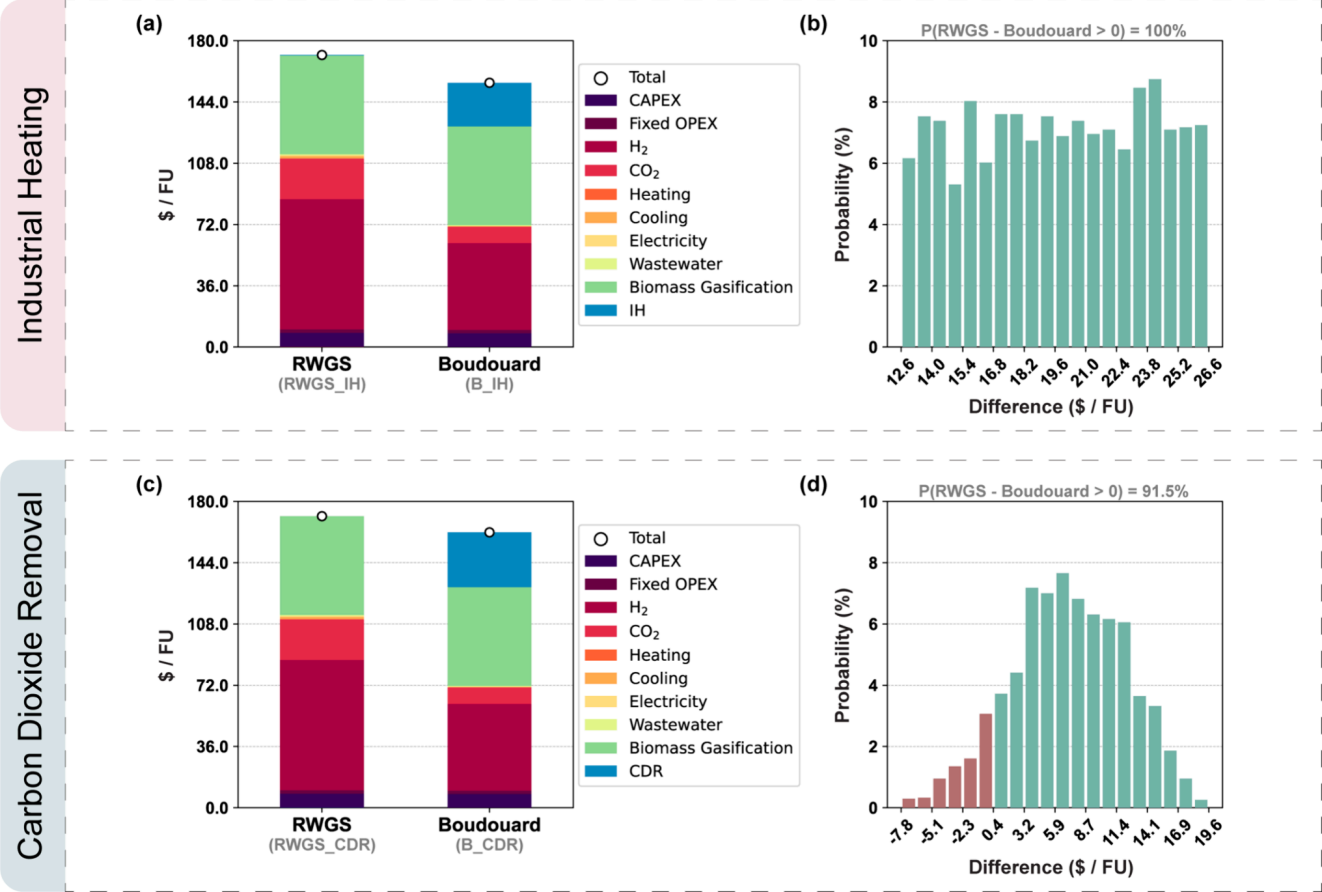
Cost breakdown and uncertainty analysis results per functional unit (FU) for RWGS and Boudouard scenarios for (a, b) the IH expanded system and (c, d) the CDR expanded system.

All in all, the same trend can be observed for both expanded systems when shifting to the Boudouard configuration: there is a decrease in the contributions of electrolytic H_2_ and DAC CO_2_, whereas the contribution of IH/CDR increases. These results suggest that a different, cheaper choice of fuel for high-temperature heat generation or a less costly carbon removal source for the Boudouard scenarios in their respective expanded systems could further improve their economic performance given that electrolytic H_2_ and DAC CO_2_ are the main cost drivers of IH and CDR, respectively. In a simplified sensitivity analysis assuming an ideal scenario of zero cost for these inputs, the Boudouard configuration achieves a cost advantage of over 20% relative to that of the RWGS configuration. While this value reflects a theoretical upper bound, it illustrates the potential for cost reduction by input replacement.

For all scenarios and expanded systems, the main contributors are wind-based electrolytic H_2_ for FT electrofuel synthesis (45% for RWGS_IH and RWGS_CDR, 33% for B_IH, and 32% for B_CDR) and biochar production as a byproduct in biomass gasification (34% for RWGS_IH and RWGS_CDR, 37% for B_IH, and 36% for B_CDR). Furthermore, the CAPEX and, consequently, the fixed OPEX contributions are similar for the RWGS and Boudouard configurations, with, respectively, 5% and 1% for both the IH and the CDR scenarios. It is worth noting that, for the biomass gasification, IH, and CDR, the CAPEX, variable OPEX, and fixed OPEX contributions were aggregated into a single contribution in the analysis. A more detailed cost breakdown of each process can be found in Section E of the Supporting Information.

The uncertainty assessment for the IH expanded system (Figure 5b) confirms that the Boudouard scenario performs better economically than the RWGS scenario regardless of the price of electrolytic H_2_ and DAC CO_2_, assuming sensible ranges for both. Notably, the cost difference between scenarios, computed for each Monte Carlo sample in the uncertainty analysis, follows a uniform distribution (Figure 5b). This is because the cost difference is driven almost entirely by the price of DAC CO_2_, which is itself sampled from a uniform distribution, as mentioned in the Methods section. Since the industrial heat generation via H_2_ combustion requires around the same amount of H_2_ as it is saved in the fuel synthesis when shifting from the RWGS reaction to the reverse Boudouard reaction, the cost of electrolytic H_2_ has virtually no impact on the cost difference between scenarios for this expanded system.

Conversely, for the CDR expanded system (Figure 5d), the cost difference depends on three factors, namely, the price of electrolytic H_2_ and DAC CO_2,_ and the stable carbon fraction of biochar. For this reason, we observe a Gaussian-like distribution for the cost variation between the RWGS and the Boudouard scenarios. Although the B_CDR scenario does not outperform the RWGS_CDR scenario for all of the price combinations, the results show a probability of over 90% cost reduction when deploying the reverse Boudouard reaction instead of the RWGS.

Overall, the uncertainty analysis results also indicate that fluctuations in the raw material prices affect the cost gap between the RWGS and Boudouard scenarios. Similarly, the stable fraction of biochar considered to calculate the abatement potential of BCR and, consequently, the equivalent amount of DACCS also influences the cost difference that can be observed between scenarios.

Although the Boudouard configuration is cheaper than the RWGS alternative, the integrated facility would still be economically unappealing compared to conventional fossil fuels. Lowering the price of key inputs—most notably of electrolytic H_2_—could narrow this gap, potentially helping FT electrofuels achieve competitive market prices.^23^ Nevertheless, other implementation limitations and challenges should be acknowledged for scale-up. First, the reverse Boudouard reaction requires high temperatures to produce CO,^44^ which raises energy consumption issues and could require specific expensive materials for the equipment as well as costly safety measures to deal with such extreme conditions. The availability of biochar and its handling could also be challenging for large-scale production, given the large volume of biochar required and its alternative competitive uses. Finally, biochar-derived syngas should undergo cleaning to remove impurities such as tar and particulates to meet syngas purity requirements of downstream catalytic processes, which could add more complexity to the design and further increase the cost.^45^

## Conclusions

In this work, we compared two process configurations for the synthesis of FT electrofuels: one using the RWGS reaction and another using the reverse Boudouard reaction in the CO_2_ to CO reduction step. The latter represents, to the best of the authors’ knowledge, the first application of this reaction in the context of FT electrofuel production. In our proposed process configuration, a new reaction system, namely, the reverse Boudouard reaction, would be added before the main FT synthesis section of current or planned projects for FT electrofuel production, whereas the rest of the plant facility would be kept the same. We used process simulation, LCA, and techno-economic assessment to evaluate the economic and environmental performance of such configurations following an expanded system approach. We included two alternative uses for biochar or its replacement for the system expansion (IH and CDR), totaling four scenarios. We considered captured CO_2_, wind-based electrolytic H_2,_ and biochar obtained as a byproduct in biomass gasification for fuel synthesis.

As expected, we found that integrating the Boudouard reaction with the FT synthesis reduces the H_2_ requirements for the FT electrofuel production, although the overall H_2_ input depends on the expanded system considered. With the shift from the RWGS route to the Boudouard route, there is a reduction in both cost and carbon footprint, which are slightly larger in the IH expanded system (10% and 11%, respectively) than in the CDR one (6% and 10%, respectively). It is worth noting that a different, cheaper choice of fuel for high-temperature heat generation or CDR technology for the Boudouard scenarios in their respective expanded systems could further improve the economic results, though possibly at the expense of worsening the environmental performance. Additionally, the results indicate that the price of raw materials and the stable carbon fraction of biochar affect the cost gap between the RWGS and the Boudouard scenarios.

Regarding the damage assessment metrics, the results indicate that within the IH expanded system, the Boudouard scenario presents a lower impact on human health, ecosystems quality, and resource depletion, with impact reductions in the range of 10–17%. On the other hand, in the expanded system with CDR, the shift from RWGS to Boudouard leads to a reduction in the damage to human health (28%) and ecosystems quality (12%) at the cost of burden shifting toward resource scarcity, with an impact increase of 6%.

All in all, this work sheds light on the potential benefits of process integration. By exploiting synergies such as increased material efficiency, we can obtain significant gains in terms of economic and environmental performance. Furthermore, our results allude to the importance of the choice of system expansion in assessing potential environmental and economic trade-offs. Varying system boundaries can lead to different assessment outcomes, which need to be evaluated carefully to quantify the potential benefits of alternative routes.

## Supplementary Material



## Notes

Carbon abatement potential calculations for BCR and DACCS (Section A), results of the simulations (Section B), economic assessment parameters and methodology (Section C), environmental assessment results (Section D), and economic assessment results (Section E) (PDF)

## ABBREVIATIONS

BCR: biochar carbon removal
CAPEX: capital expenditures
CCS: carbon capture and storage
CDR: carbon dioxide removal
CEPCI: chemical engineering plant cost index
DAC: direct air capture
DACCS: direct air carbon capture and storage
FT: Fischer–Tropsch
GWI: global warming impact
IH: industrial heating
LCA: life cycle assessment
LCI: life cycle inventory
OPEX: operational expenditures
PSA: pressure swing adsorption
RWGS: reverse water–gas shift

## References

[1] International Energy Agency (IEA). Transport. https://www.iea.org/energy-system/transport (accessed 2025–02–17).

[2] International Energy Agency (IEA). Energy consumption in transport by fuel in the Net Zero Scenario, 1975–2030. https://www.iea.org/data-and-statistics/charts/energy-consumption-in-transport-by-fuel-in-the-net-zero-scenario-1975-2030 (accessed 2025–02–18).

[3] Peters R., Wegener N., Samsun R. C., Schorn F., Riese J., Grünewald M., Stolten D. (2022). A Techno-Economic Assessment of Fischer–Tropsch
Fuels Based on Syngas from Co-Electrolysis. Processes.

[4] Ausfelder F., Wagemann K. (2020). Power-to-Fuels: E-Fuels as an Important Option for
a Climate-Friendly Mobility of the Future. Chem.
Ing. Technol..

[5] International Renewable Energy Agency (IRENA). Decarbonising Hard-to-Abate Sectors with Renewables: Perspectives for the G7; IRENA 2024

[6] Dieterich V., Buttler A., Hanel A., Spliethoff H., Fendt S. (2020). Power-to-Liquid *via* Synthesis of Methanol, DME or
Fischer–Tropsch-Fuels: A Review. Energy
Environ. Sci..

[7] Saeidi S., Talebi Amiri M., Saidina Amin N. A., Rahimpour M. R. (2014). Progress
in Reactors for High-Temperature Fischer–Tropsch Process: Determination
Place of Intensifier Reactor Perspective. Int.
J. Chem. React. Eng..

[8] Daza Y. A., Kuhn J. N. (2016). CO2 Conversion by Reverse Water Gas Shift Catalysis:
Comparison of Catalysts, Mechanisms and Their Consequences for CO_2_ Conversion to Liquid Fuels. RSC Adv..

[9] Atsonios K., Panopoulos K. D., Kakaras E. (2016). Investigation of Technical and Economic
Aspects for Methanol Production through CO_2_ Hydrogenation. Int. J. Hydrog. Energy.

[10] González-Garay A., Frei M. S., Al-Qahtani A., Mondelli C., Guillén-Gosálbez G., Pérez-Ramírez J. (2019). Plant-to-Planet Analysis of CO_2_ -Based Methanol Processes. Energy Environ.
Sci..

[11] Pérez-Fortes M., Schöneberger J. C., Boulamanti A., Tzimas E. (2016). Methanol Synthesis Using Captured CO_2_ as
Raw Material: Techno-Economic and Environmental Assessment. Appl. Energy.

[12] D’Angelo S. C., Cobo S., Tulus V., Nabera A., Martín A. J., Pérez-Ramírez J., Guillén-Gosálbez G. (2021). Planetary
Boundaries Analysis of Low-Carbon Ammonia Production Routes. ACS Sustainable Chem. Eng..

[13] Ioannou I., D’Angelo S. C., Martín A. J., Pérez-Ramírez J., Guillén-Gosálbez G. (2020). Hybridization of Fossil- and CO2-Based
Routes for Ethylene Production Using Renewable Energy. ChemSusChem.

[14] König D. H., Freiberg M., Dietrich R.-U., Wörner A. (2015). Techno-Economic
Study of the Storage of Fluctuating Renewable Energy in Liquid Hydrocarbons. Fuel.

[15] Medrano-García J. D., Charalambous M. A., Guillén-Gosálbez G. (2022). Economic and
Environmental Barriers of CO_2_-Based Fischer–Tropsch
Electro-Diesel. ACS Sustainable Chem. Eng..

[16] Freire
Ordóñez D., Halfdanarson T., Ganzer C., Shah N., Dowell N. M., Guillén-Gosálbez G. (2022). Evaluation
of the Potential Use of E-Fuels in the European Aviation Sector: A
Comprehensive Economic and Environmental Assessment Including Externalities. Sustain. Energy Fuels.

[17] Brynolf S., Taljegard M., Grahn M., Hansson J. (2018). Electrofuels for the
Transport Sector: A Review of Production Costs. Renew. Sustain. Energy Rev..

[18] Kim J., Guo W., Kim H., Choe S., Kim S. Y., Ahn S. H. (2022). Gaseous
CO_2_ Electrolysis: Progress, Challenges, and Prospects. ACS Sustainable Chem. Eng..

[19] Jouny M., Luc W., Jiao F. (2018). General Techno-Economic
Analysis of CO_2_ Electrolysis
Systems. Ind. Eng. Chem. Res..

[20] Inuzuka R., Osada N., Tsuchiya N., Kawamori H., Ichikawa N., Ishihara T. (2025). CO_2_/H_2_O Co-Electrolysis Using
Single and Stack Solid Oxide Electrolysis Cell (SOEC). Electrochim. Acta.

[21] Hou X., Jiang Y., Wei K., Jiang C., Jen T.-C., Yao Y., Liu X., Ma J., Irvine J. T. S. (2024). Syngas Production
from CO_2_ and H_2_O via Solid-Oxide Electrolyzer
Cells: Fundamentals, Materials, Degradation, Operating Conditions,
and Applications. Chem. Rev..

[22] Safaei H., Aziz M. J. (2017). Thermodynamic Analysis
of Three Compressed Air Energy
Storage Systems: Conventional, Adiabatic, and Hydrogen-Fueled. Energies.

[23] Medrano-García J. D., Chagas M. T., Guillén-Gosálbez G. (2025). Integrating
the Reverse Boudouard Reaction for a More Efficient Green Methanol
Synthesis from CO_2_ and Renewable Energy. ACS Sustainable Chem. Eng..

[24] Alsawadi A. M., Marsh R., Steer J. M., Morgan D. (2025). A Study of the Mechanisms
Associated with CO_2_ Utilisation via the Reverse Boudouard
Reaction. Fuel.

[25] Intergovernmental Panel on Climate Change (IPCC). 2019 Refinement to the 2006 IPCC Guidelines for National Greenhouse Gas Inventories; 2019. https://www.ipcc-nggip.iges.or.jp/public/2019rf/pdf/4_Volume4/19R_V4_Ch02_Ap4_Biochar.pdf (accessed 2025–02–03).

[26] Joshua
Abioye K., Rajamanickam R., Ogunjinmi T., Paul S., Selvasembian R., Ighalo J. O. (2025). Advancements in
Biomass Waste Conversion to Sustainable Biofuels via Gasification. Chem. Eng. J..

[27] Peters J. F., Iribarren D., Dufour J. (2015). Biomass Pyrolysis for
Biochar or
Energy Applications? A Life Cycle Assessment. Environ. Sci. Technol..

[28] You S., Ok Y. S., Chen S. S., Tsang D. C. W., Kwon E. E., Lee J., Wang C.-H. (2017). A Critical
Review on Sustainable Biochar System through
Gasification: Energy and Environmental Applications. Bioresour. Technol..

[29] Liu H., Tang Y., Ma X., Tang J., Deng J., Yue W. (2025). Calcium Looping-Enhanced
Biomass Gasification for Methanol Production:
Integrating Methane Dry Reforming and Carbon Utilization. Sep. Purif. Technol..

[30] Medrano-García J. D., Calvo-Serrano R., Tian H., Guillén-Gosálbez G. (2025). Win-Win More
Sustainable Routes for Acetic Acid Synthesis. ACS Sustainable Chem. Eng..

[31] Hunt J., Ferrari A., Lita A., Crosswhite M., Ashley B., Stiegman A. E. (2013). Microwave-Specific
Enhancement of
the Carbon-Carbon Dioxide (Boudouard) Reaction. J. Phys. Chem. C.

[32] Hillestad M. (2015). Modeling the
Fischer–Tropsch Product Distribution and Model Implementation. Chem. Prod. Process Model..

[33] Mohanty S., Saraf D. N., Kunzru D. (1991). Modeling of
a Hydrocracking Reactor. Fuel Process. Technol..

[34] Bhutani N., Ray A. K., Rangaiah G. P. (2006). Modeling, Simulation,
and Multi-Objective
Optimization of an Industrial Hydrocracking Unit. Ind. Eng. Chem. Res..

[35] Delgado H. E., Cappello V., Zang G., Sun P., Ng C., Vyawahare P., Elgowainy A. A., Wendt D. S., Boardman R. D., Marcinkoski J. (2023). Techno-Economic
Analysis and Life Cycle Analysis of
e-Fuel Production Using Nuclear Energy. J. CO2
Util..

[36] Atsonios K., Li J., Inglezakis V. J. (2023). Process
Analysis and Comparative
Assessment of Advanced Thermochemical Pathways for E-Kerosene Production. Energy.

[37] van
Zelm R., Hennequin T., Huijbregts M. A. J. (2025). Performing Life Cycle Impact Assessment
with the Midpoint and Endpoint Method ReCiPe. Nat. Protoc..

[38] Schumacher, L. ReCiPe. PRé Sustainability. https://pre-sustainability.com/articles/recipe/ (accessed 2025–07–29).

[39] Sinnott, R. ; Towler, G. Costing and Project Evaluation. In Chemical Engineering Design; Elsevier, 2020; pp 275–369. 10.1016/B978-0-08-102599-4.00006-0.

[40] Onel O., Niziolek A. M., Elia J. A., Baliban R. C., Floudas C. A. (2015). Biomass
and Natural Gas to Liquid Transportation Fuels and Olefins (BGTL+C2_C4):
Process Synthesis and Global Optimization. Ind.
Eng. Chem. Res..

[41] Nabera A., José Martín A., Istrate R., Pérez-Ramírez J., Guillén-Goślbez G. (2024). Integrating Climate Policies in the
Sustainability Analysis of Green Chemicals. Green Chem..

[42] Spath, P. ; Aden, A. ; Eggeman, T. ; Ringer, M. ; Wallace, B. ; Jechura, J. Biomass to Hydrogen Production Detailed Design and Economics Utilizing the Battelle Columbus Laboratory Indirectly-Heated Gasifier; NREL/TP-510–37408; National Renewable Energy Lab.(NREL) 2005. 10.2172/15016221

[43] Salah C., Istrate R., Bjo̷rn A., Tulus V., Pérez-Ramírez J., Guillén-Gosálbez G. (2024). Environmental Benefits of Circular
Ethylene Production from Polymer Waste. ACS
Sustainable Chem. Eng..

[44] Lahijani P., Zainal Z. A., Mohammadi M., Mohamed A. R. (2015). Conversion of the
Greenhouse Gas CO_2_ to the Fuel Gas CO via the Boudouard
Reaction: A Review. Renew. Sustain. Energy Rev..

[45] Hrbek, J. ; Pfeifer, C. ; Blanco-Sanchez, P. ; Baldwin, R. ; Swanson, M. ; Strege, J. Gas Cleaning from Gasification for Production of Biofuels and Biochemicals; IEA Bioenergy, 2025.

