# Supplementary material for: Biochar Enhances Fischer–Tropsch Electrofuels from CO2 and Renewable Energy
Source: ACS Sustain Chem Eng. 2025 Oct 23;13(43):18424–34. doi: 10.1021/acssuschemeng.5c03540 (PMC12587439; doi:10.1021/acssuschemeng.5c03540)
Supplement: Supplementary file 1 [file sc5c03540_si_001.pdf]

Supporting Information

Biochar enhances Fischer-Tropsch electrofuels from CO_2 and renewable energy

Marina T. Chagas[†], Juan D. Medrano-García[†], and Gonzalo Guillén-Gosálbez[,†]*

[†] Institute for Chemical and Bioengineering, Department of Chemistry and Applied Biosciences, ETH Zurich, Vladimir Prelog Weg 1, 8093 Zurich, Switzerland.

[*] Corresponding author: gonzalo.guillen.gosalbez@chem.ethz.ch.

This document includes additional material to the content presented in the main article. Here we report the carbon abatement potential calculations, the life cycle inventories, the economic assessment parameters and methodology, and further environmental and economic results.

Number of pages: 28

Number of figures: 0

Number of tables: 33

A. Carbon Abatement Calculation

A1. Biochar Carbon Removal (BCR)

The carbon abatement potential of biochar carbon removal (BCR) (BCR_{net}, in kg CO_2-eq / kg biochar) was determined according to **Eq. (S1)** from the ratio of molar weights of carbon dioxide (CO_2) and biochar (MW_{CO_2} and $MW_{biochar}$, respectively), assuming a carbon content (f_c) of biochar of 100%[1] and the stable carbon fraction (f_s) in the biochar of 0.89[2], while neglecting lifecycle emissions. As biochar is assumed to be pure carbon, $MW_{biochar}$ is 12 kg/kmol.

$$BCR_{net} = \frac{MW_{CO_2}}{MW_{biochar}} \cdot f_c \cdot f_s \quad \text{(S1)}$$

From **Eq. (S1),** we found that BCR_{net} is 3.26 kg CO_2-eq / kg biochar. Accordingly, **Table S1** displays the life cycle inventory (LCI) of BCR given 1 kg of stored biochar as the functional unit.

Table S1. LCI for BCR (3.26 kg CO_2-eq/kg of stored biochar).

**Functional unit: 1 kg of stored biochar**		
**Input**	**Amount**	**Units**
Biochar (from biomass gasification)[1]	1.00	kg
**Output**		
Stored biochar (from biomass gasification)	1.00	kg

The total carbon abatement potential for BCR (BCR_{total}) was then calculated according to **Eq. (S2),** considering the biochar output from the biomass gasification process per functional unit ($M_{biochar}$).

$$BCR_{total} = BCR_{net} \cdot M_{biochar} \quad (S2)$$

Thus, for our expanded system with BCR as the carbon dioxide removal technology (CDR), namely RWGS_CDR scenario, BCR_{total} was determined to be 34.8 kg CO_2-eq per functional unit.

A2. Direct Air Carbon Capture and Storage (DACCS)

In order to obtain the same climate change mitigation potential as BCR_{net}, the amount of direct air-captured (DAC) CO_2 to be stored was calculated considering the carbon abatement potential of direct air carbon capture and storage (DACCS) ($DACCS_{net}$, in kg CO_2-eq / kg DAC-CO_2).

To this end, $DACCS_{net}$ was estimated considering that CO_2 from DAC, originally at 25 °C and 1 bar, is compressed to 110 bar in four stages with intercooling to 40 °C. The resulting life cycle inventory (LCI) of the DACCS process, for a functional unit of 1 kg of stored CO_2 from DAC, is displayed in **Table S2**. The inventory of CO_2 from DAC was taken from Keith *et al.*[3] and adapted by Medrano-García *et al.*[4].

Table S2. LCI for DACCS (0.5 kg CO_2-eq / kg of stored CO_2 from DAC)..

**Functional unit: 1 kg of stored CO_2 from DAC**		
**Input**	**Amount**	**Units**
Carbon dioxide (from direct air capture)[3,4]	1.00	kg
Cooling (water 20 to 25 ºC)[5]	$6.02\cdot10^{-1}$	MJ
Electricity (high voltage)	$1.13\cdot10^{-1}$	kWh
**Output**		
Stored carbon dioxide (from direct air capture)	1.00	kg

$DACCS_{net}$ was determined with the assessment method ReCiPe 2016 v1.03 Midpoint (H) by taking the absolute value of the global warming midpoint and was found to be 0.50 kg CO_2-eq / kg DAC-CO_2.

Finally, the input of CO_2 from DAC for storage per functional unit ($M_{DAC-CO_{2}input}$) was calculated according to **Eq. (S3)**.

$$M_{DAC-CO_{2}input} = \frac{BCR_{total}}{DACCS_{net}} \tag{S3}$$

Thus, for our expanded system with DACCS as the CDR technology, namely B_CDR scenario, $M_{DAC-CO_{2}input}$ was determined to be 69.5 kg per functional unit.

B. Simulation Results

In this section, we present the main results for the reverse water-gas shift (RWGS) and Boudouard configurations for Fischer-Tropsch (FT) electrofuels production (**Tables S3** and **S4**, respectively). Each LCI contains the net material and energy flows per functional unit of product, namely, 1 GJ of electrofuel.

Additionally, the inventories for BCR and DACCS considering a functional unit of 1 kg of CO_2-eq stored can be found respectively in **Tables S5** and **S6**. The change of functional unit in contrast to **Tables S1** and **S2** was made to facilitate the depiction and comparison of the scenarios within each expanded system, to be done subsequently.

Tables S7-S10 display the LCIs for each of the four scenarios (RWGS_IH, B_IH, RWGS_CDR, and B_CDR), considering the system expansion approach. The simulation results for the biomass gasification for hydrogen (H_2) production with carbon capture and storage (CCS), where biochar is obtained as a byproduct, and the industrial heat generation via biochar or H_2 combustion were obtained from Medrano-García *et al.*[1]

Table S3. LCI for RWGS configuration.

**Functional unit: 1 GJ of electrofuel**		
**Input**	**Amount**	**Units**
Hydrogen (from wind electrolysis)[6]	11.12	kg
Carbon dioxide (from direct air capture)[3,4]	66.00	kg
Cooling (water 20 to 25 ºC)[5]	$4.05 \cdot 10^{3}$	MJ
Electricity (high voltage)	6.08	kWh
**Output**		
Electrofuel	1.00	GJ
Water (emission to water)	$3.13 \cdot 10^{-1}$	m^3

Table S4. LCI for Boudouard configuration.

Functional unit: 1 GJ of electrofuel		
**Input**	**Amount**	**Units**
Hydrogen (from wind electrolysis)[6]	7.40	kg
Carbon dioxide (from direct air capture)[3,4]	26.82	kg
Biochar (from biomass gasification)[1]	$1.07 \cdot 10^{1}$	kg
Heating (from natural gas)	39.96	MJ
Cooling (water 20 to 25 ºC)[5]	$4.51 \cdot 10^{2}$	MJ
Electricity (high voltage)	3.17	kWh
**Output**		
Electrofuel	1.00	GJ
Water (emission to water)	$2.12 \cdot 10^{-1}$	m^3

Table S5. LCI for BCR.

Functional unit: 1 kg of stored CO_2-eq from biochar		
**Input**	**Amount**	**Units**
Biochar (from biomass gasification)[1]	$3.06 \cdot 10^{-1}$	kg
**Output**		
Stored CO_2-eq	1.00	kg

Table S6. LCI for DACCS.

Functional unit: 1 kg of stored CO_2-eq from DACCS		
**Input**	**Amount**	**Units**
Carbon dioxide (from direct air capture)[3,4]	2.00	kg
Cooling (water 20 to 25 ºC)[5]	1.20	MJ
Electricity (high voltage)	$2.27 \cdot 10^{-1}$	kWh
**Output**		
Stored CO_2-eq	1.00	kg

Table S7. LCI for the RWGS scenario within the industrial heating (IH) expanded system (i.e., RWGS_IH).

Functional unit: 1 GJ of electrofuel, 24.4 kg of biogenic H_2, and 287.3 MJ of high temperature heat		
**Input**	**Amount**	**Units**
FT electrofuels production (**Table S3**)	1.00	GJ
Heating (from biochar)[1]*	$2.87 \cdot 10^{2}$	MJ

*obtaining biochar via biomass gasification implies the production of 24.4 kg of biogenic H_2

Table S8. LCI for the Boudouard scenario within the IH expanded system (i.e., B_IH).

Functional unit: 1 GJ of electrofuel, 24.4 kg of biogenic H_2, and 287.3 MJ of high temperature heat		
**Input**	**Amount**	**Units**
FT electrofuels production (**Table S4**)*	1.00	GJ
Heating (from H_2)[1]	$2.87 \cdot 10^2$	MJ

*obtaining biochar via biomass gasification implies the production of 24.4 kg of biogenic H_2

Table S9. LCI for RWGS scenario within the CDR expanded system (i.e., RWGS_CDR).

Functional unit: 1 GJ of electrofuel, 24.4 kg of biogenic H_2, and 34.8 kg of stored CO_2-eq		
**Input**	**Amount**	**Units**
FT electrofuels production (**Table S3**)	1.00	GJ
BCR (**Table S5**)*	$3.48 \cdot 10^1$	kg

*obtaining biochar via biomass gasification implies the production of 24.4 kg of biogenic H_2

Table S10. LCI for the Boudouard scenario within the CDR expanded system (i.e., B_CDR)

**Functional unit: 1 GJ of electrofuel, 24.4 kg of biogenic H_2, and 34.8 kg of stored CO_2-eq**		
**Input**	**Amount**	**Units**
FT electrofuels production (**Table S4**)*	1.00	GJ
DACCS (**Table S6**)	$3.48 \cdot 10^{1}$	kg

*obtaining biochar via biomass gasification implies the production of 24.4 kg of biogenic H_2

C. Economic Assessment

In this section, we present the economic data used in the analysis and the capital investment cost methodology employed, as well as the parameters for the uncertainty assessment.

C.1. Economic Parameters

Table S11. Raw material and utility prices.

Input	Amount	Units	Reference
H_2 (from wind electricity)	$6.88 \cdot 10^{0}$	\$/kg	[7]
CO_2 (from direct air capture)	$3.62 \cdot 10^{-1}$	\$/kg	[7]
Biomass	$3.00 \cdot 10^{-2}$	\$/dry kg	[1]
Heating (from natural gas)	$1.96 \cdot 10^{0}$	\$/GJ	[7]
Cooling (water from 20 to 25 ºC)	$3.78 \cdot 10^{-1}$	\$/GJ	[5]
Electricity (global mix)	$1.04 \cdot 10^{-1}$	\$/kWh	[7]
Wastewater treatment	$1.65 \cdot 10^{0}$	\$/m^3	[8]

Table S12. Parameters used for the purchased equipment cost of compressors, heat exchangers, pumps, furnace, vessels, and trays, for year 2007.[9]

Process unit	Sizing unit (S)	S_{lower}	S_{upper}	a	b	n
Centrifugal compressor	Power [kW]	75	30’000	490’000	16’800	0.60
U-tube sheel and tube	Area [m^2]	10	1’000	24’000	46	1.20
Single stage centrifugal pump	Flow [L/s]	0.2	126	6’900	206	0.9
Cylindrical furnace	Duty [MW]	0.2	60	130	440	1.80
Pressure vessel, vertical	Shell mass [kg]	160	250’000	10’000	29	0.85
Sieve tray	Diameter [m]	0.5	5	110	380	1.8

Table S13. Parameters used for the economic calculations of RWGS, FT, and hydrocracking (HC), and PSA units.

Process unit	Sizing unit	Year	PC_{ref}	S_{ref}	D
RWGS reactor[10]	Flow[t/d]	2010	$3.05 \cdot 10^6$	2’556	0.65
FT reactor[6]	Flow [m^3/h]	2005	$1.05 \cdot 10^7$	71’400	1
HC reactor[10]	Flow [kg/s]	2009	$9.37 \cdot 10^6$	1.13	0.7
PSA[11]	Purge flow [kmol/s]	2014	$7.84 \cdot 10^6$	0.29	0.65

C.2. Capital Expenditures (CAPEX)

The fixed capital costs (FCC) were estimated from the purchased equipment costs (C_e) for the main units in each process, with the exception of the reactors (RWGS, FT, and HC) and the PSA system, according to the standard procedures.[9] **Eq. (S4)** was used to calculate C_e for each unit based on the appropriate size parameter (S) and cost constants (a, b, and n). These parameters can be found in **Table S12**. When S surpassed S_{upper}, the unit was assumed to be divided into smaller units of equal size, to operate in parallel, for which S stayed within the limit. For the purchased cost calculation, the Boudouard reactor was approximated by a furnace.

The cost function and parameters of the reactors were taken from König *et al.*[10] and Medrano-García *et al.*[6]. The C_e for each unit was computed given its capacity (S) according to **Eq. (S5),** using the parameters from **Table S13**. The Chemical Engineering Plant Cost Index (CEPCI) was used to adjust the purchased cost of each reactor to the same reference year as the ones calculated via **Eq. (S4)**, namely 2007.

$$C_e = a + b \cdot S^n \tag{S4}$$

$$C_e = PC_{ref} \cdot \left(\frac{S}{S_{ref}}\right)^D \cdot \left(\frac{CEPCI_{2007}}{CEPCI_{ref}}\right) \tag{S5}$$

The inside battery limits investment (C_{IBL}) was estimated from the C_e of all units (except the PSA system) using **Eq. (S6)**, whereas the FCC was obtained from **Eq. (S7)**. The factors for both equations are displayed in **Table S14**.

$$C_{IBL} = \sum C_e \cdot \left[\left(1 + f_p\right) \cdot f_m + (f_{er} + f_{el} + f_i + f_c + f_s + f_l)\right] \tag{S6}$$

$$FCC = C_{IBL} \cdot (1 + OS) \cdot (1 + D\&E + X) \tag{S7}$$

Table S14. Factors used for the estimation of the FCC.[9]

Factor	Description	RWGS scenarios	Boudouard scenarios
f_p	Piping	0.8	0.6
f_m	Materials (stainless steel)	1.3	1.3
f_{er}	Equipment erection	0.3	0.5
f_{el}	Electrical	0.2	0.2
f_i	Instrumentation and control	0.3	0.3
f_c	Civil	0.3	0.3
f_s	Structures and buildings	0.2	0.2
f_l	Lagging and paint	0.1	0.1
OS	Offsites	0.3	0.4
$D\&E$	Design and engineering	0.3	0.25
X	Contingency	0.1	0.1

In the case of the PSA system, the cost function was taken from Onel *et al.*[11]. The purchased cost (PC) was computed given its capacity (S) according to **Eq. (S8)**, using the parameters from **Table S13**. PC_{ref} and S_{ref} are, respectively, the base unit cost and base capacity, and D is the scaling factor. Additionally, BOP is the balance of plant cost, assumed to be 20%.[11] Finally, the FCC in regard to the PSA unit was obtained via **Eq. (S9)**, assuming the indirect costs (IC) to be 32%.[11]

$$PC = PC_{ref} \cdot \left(\frac{S}{S_{ref}}\right)^{D} \cdot (1 + BOP) \tag{S8}$$

$$FCC = \sum PC \cdot (1 + IC) \quad \text{(S9)}$$

The $FCCs$ from both **Eq. (S7)** and **Eq. (S9)** were adjusted with the Chemical Engineering Plant Cost Index (CEPCI) to USD 2023, according to equation **Eq. (S10)**, and summed to yield the total FCC of the process (FCC_{total}).

$$FCC_{2023} = FCC_{year\ A} \cdot \frac{CEPCI_{2023}}{CEPCI_{year\ A}} \quad \text{(S10)}$$

The annualized capital cost (ACC) was calculated according to **Eq. (S11)** for a plant lifetime (n) of 30 years and an interest rate (i) of 10%.

$$ACC = FCC_{total} \cdot \frac{i \cdot (1 + i)^n}{(1 + i)^n - 1} \quad \text{(S11)}$$

The description of the CAPEX calculations for the biomass gasification for hydrogen (H_2) production with carbon capture and storage (CCS), where biochar is obtained as a byproduct, and for the industrial heat generation via biochar or H_2 combustion can be found in Medrano-García *et al.*[1]

C.3. Operational Expenditures (OPEX)

The OPEX were obtained as the sum of the fixed and variable contributions ($OPEX_{fix}$ and $OPEX_{var}$, respectively). $OPEX_{var}$ accounts for the raw materials and utilities and was calculated based on the purchase prices (**Table S11**) and inputs to the process per functional unit. $OPEX_{fix}$, on the other hand, accounts for expenses related to labor, maintenance, taxes, insurance, land, and plant overheads.31. It was derived from the CAPEX following the methodology by Sinnott and Towler with the factors from **Table S15**, as described by **Eqs. (S12)-(S20).**[9]

Table S15. Factors used for the estimation of the $OPEX_{fix}$.[9]

Factors		Value
Operator salary ($/year)[1]	$salary$	50'000
Number of shift positions[1]	n_{shifts}	3
Number of operators per shift[1]	$n_{operators}$	4.8
Supervision	$f_{supervision}$	25%
Direct salary overhead	f_{salary}	40%
Maintenance	$f_{maintenance}$	3%
Property taxes and insurance	$f_{property}$	1%
Rent of land	f_{rent}	1%
General plant overhead	f_{plant}	65%

$$operating\ labor = salary \cdot n_{shifts} \cdot n_{operators} \tag{S12}$$

$$supervision = f_{supervision} \cdot operating\ labor \tag{S13}$$

$$direct\ salary\ overhead = f_{salary} \cdot (operating\ labor + supervision) \tag{S14}$$

$$labor\ costs = operating\ labor + supervision + direct\ salary\ overhead \tag{S15}$$

$$maintenance = f_{maintenance} \cdot C_{IBL} \tag{S16}$$

$$taxes\ \&\ insurance = f_{property} \cdot C_{IBL} \tag{S17}$$

$$rent = f_{rent} \cdot C_{IBL} \cdot (1 + OS) \tag{S18}$$

$$plant\ overhead = f_{plant} \cdot (labor\ costs + maintenance) \tag{S19}$$

$$OPEX_{fix} = labor + maintenance + taxes\ \&\ insurance + rent + plant\ overhead \tag{S20}$$

C.4. Total Cost

For each scenario, the total cost was computed considering the ACC, $OPEX_{var}$, and $OPEX_{fix}$ contributions for the FT electrofuels production, the biochar production, and the alternative biochar application or its replacement. A cost breakdown per functional unit for each scenario was obtained considering an annual operation of 8000 h.

C.5. Parameters for Uncertainty Assessment

Table S16. Raw material and utility prices.

Input	Low	High	Units	Reference
H_2 (from wind electricity)	$5.23 \cdot 10^{0}$	$8.48 \cdot 10^{0}$	\$/kg	[7]
CO_2 (from direct air capture)	$2.69 \cdot 10^{-1}$	$6.23 \cdot 10^{-1}$	\$/kg	[7]
Stable carbon fraction of biochar	$8.90 \cdot 10^{-1}$	$1.00 \cdot 10^{0}$	-	[2]

D. Life Cycle Assessment (LCA) Results

In this section, we present the full environmental assessment results for the four studied scenarios. All results are given per functional unit of the corresponding scenario.

D.1. Midpoint results

Table S17. ReCiPe 2016 v1.03 midpoint (H) results for the RWGS scenario within the IH expanded system (i.e., RWGS_IH).

Impact category	Unit	Total	FT electrofuels	Biomass gasification	High temperature heating
Global warming	kg CO_2-eq	$-2.08\cdot10^{2}$	$-6.50\cdot10^{0}$	$-2.41\cdot10^{2}$	$3.91\cdot10^{1}$
Stratospheric ozone depletion	kg CFC11-eq	$8.08\cdot10^{-5}$	$2.20\cdot10^{-5}$	$5.87\cdot10^{-5}$	0.00
Ionizing radiation	kBq Co-60-eq	$1.94\cdot10^{1}$	$3.66\cdot10^{0}$	$1.57\cdot10^{1}$	0.00
Ozone formation, Human health	kg NOx-eq	$5.28\cdot10^{-1}$	$1.16\cdot10^{-1}$	$4.12\cdot10^{-1}$	0.00
Fine particulate matter formation	kg PM2.5-eq	$4.63\cdot10^{-1}$	$1.08\cdot10^{-1}$	$3.55\cdot10^{-1}$	0.00
Ozone formation, Terrestrial ecosystems	kg NOx-eq	$5.37\cdot10^{-1}$	$1.20\cdot10^{-1}$	$4.17\cdot10^{-1}$	0.00
Terrestrial acidification	kg SO_2-eq	$9.28\cdot10^{-1}$	$2.51\cdot10^{-1}$	$6.77\cdot10^{-1}$	0.00
Freshwater eutrophication	kg P-eq	$1.27\cdot10^{-1}$	$4.60\cdot10^{-2}$	$8.13\cdot10^{-2}$	0.00
Marine eutrophication	kg N-eq	$1.06\cdot10^{-2}$	$4.95\cdot10^{-3}$	$5.67\cdot10^{-3}$	0.00
Terrestrial ecotoxicity	kg 1,4-DCB	$8.89\cdot10^{2}$	$7.48\cdot10^{2}$	$1.42\cdot10^{2}$	0.00
Freshwater ecotoxicity	kg 1,4-DCB	$4.38\cdot10^{1}$	$3.93\cdot10^{1}$	$4.58\cdot10^{0}$	0.00
Marine ecotoxicity	kg 1,4-DCB	$5.46\cdot10^{1}$	$4.84\cdot10^{1}$	$6.27\cdot10^{0}$	0.00
Human carcinogenic toxicity	kg 1,4-DCB	$1.26\cdot10^{1}$	$6.83\cdot10^{0}$	$5.77\cdot10^{0}$	0.00
Human non-carcinogenic toxicity	kg 1,4-DCB	$4.55\cdot10^{2}$	$2.28\cdot10^{2}$	$2.26\cdot10^{2}$	0.00
Land use	m^2a crop-eq	$1.38\cdot10^{2}$	$1.45\cdot10^{0}$	$1.36\cdot10^{2}$	0.00
Mineral resource scarcity	kg Cu-eq	$9.18\cdot10^{-1}$	$8.23\cdot10^{-1}$	$9.51\cdot10^{-2}$	0.00
Fossil resource scarcity	kg oil-eq	$5.79\cdot10^{1}$	$1.58\cdot10^{1}$	$4.21\cdot10^{1}$	0.00
Water consumption	m^3	$5.08\cdot10^{0}$	$2.10\cdot10^{0}$	$3.02\cdot10^{0}$	$-4.72\cdot10^{-2}$

Table S18. ReCiPe 2016 v1.03 midpoint (H) results for the Boudouard scenario within the IH expanded system (i.e., B_IH).

Impact category	Unit	Total	FT electrofuels	Biomass gasification	High temperature heating
Global warming	kg CO_2-eq	$-2.30\cdot10^{2}$	$3.17\cdot10^{0}$	$-2.41\cdot10^{2}$	$7.37\cdot10^{0}$
Stratospheric ozone depletion	kg CFC11-eq	$7.55\cdot10^{-5}$	$1.24\cdot10^{-5}$	$5.87\cdot10^{-5}$	$4.46\cdot10^{-6}$
Ionizing radiation	kBq Co-60-eq	$1.79\cdot10^{1}$	$1.70\cdot10^{0}$	$1.57\cdot10^{1}$	$5.08\cdot10^{-1}$
Ozone formation, Human health	kg NOx-eq	$4.98\cdot10^{-1}$	$6.35\cdot10^{-2}$	$4.12\cdot10^{-1}$	$2.26\cdot10^{-2}$
Fine particulate matter formation	kg PM2.5-eq	$4.37\cdot10^{-1}$	$5.93\cdot10^{-2}$	$3.55\cdot10^{-1}$	$2.32\cdot10^{-2}$
Ozone formation, Terrestrial ecosystems	kg NOx-eq	$5.06\cdot10^{-1}$	$6.55\cdot10^{-2}$	$4.17\cdot10^{-1}$	$2.34\cdot10^{-2}$
Terrestrial acidification	kg SO_2-eq	$8.72\cdot10^{-1}$	$1.41\cdot10^{-1}$	$6.77\cdot10^{-1}$	$5.36\cdot10^{-2}$
Freshwater eutrophication	kg P-eq	$1.21\cdot10^{-1}$	$2.75\cdot10^{-2}$	$8.13\cdot10^{-2}$	$1.22\cdot10^{-2}$
Marine eutrophication	kg N-eq	$9.58\cdot10^{-3}$	$3.09\cdot10^{-3}$	$5.67\cdot10^{-3}$	$8.24\cdot10^{-4}$
Terrestrial ecotoxicity	kg 1,4-DCB	$8.72\cdot10^{2}$	$4.91\cdot10^{2}$	$1.42\cdot10^{2}$	$2.40\cdot10^{2}$
Freshwater ecotoxicity	kg 1,4-DCB	$4.34\cdot10^{1}$	$2.60\cdot10^{1}$	$4.58\cdot10^{0}$	$1.28\cdot10^{1}$
Marine ecotoxicity	kg 1,4-DCB	$5.41\cdot10^{1}$	$3.20\cdot10^{1}$	$6.27\cdot10^{0}$	$1.58\cdot10^{1}$
Human carcinogenic toxicity	kg 1,4-DCB	$1.19\cdot10^{1}$	$4.18\cdot10^{0}$	$5.77\cdot10^{0}$	$1.94\cdot10^{0}$
Human non-carcinogenic toxicity	kg 1,4-DCB	$4.46\cdot10^{2}$	$1.48\cdot10^{2}$	$2.26\cdot10^{2}$	$7.13\cdot10^{1}$
Land use	m^2a crop-eq	$1.37\cdot10^{2}$	$8.35\cdot10^{-1}$	$1.36\cdot10^{2}$	$3.24\cdot10^{-1}$
Mineral resource scarcity	kg Cu-eq	$8.96\cdot10^{-1}$	$5.38\cdot10^{-1}$	$9.51\cdot10^{-2}$	$2.63\cdot10^{-1}$
Fossil resource scarcity	kg oil-eq	$5.19\cdot10^{1}$	$8.01\cdot10^{0}$	$4.21\cdot10^{1}$	$1.77\cdot10^{0}$
Water consumption	m^3	$3.50\cdot10^{0}$	$3.81\cdot10^{-1}$	$3.02\cdot10^{0}$	$9.48\cdot10^{-2}$

Table S19. ReCiPe 2016 v1.03 midpoint (H) results for the RWGS scenario within the CDR expanded system (i.e., RWGS_CDR).

Impact category	Unit	Total	FT electrofuels	Biomass gasification	Carbon dioxide removal
Global warming	kg CO_2-eq	$-2.47 \cdot 10^{2}$	$-6.50 \cdot 10^{0}$	$-2.41 \cdot 10^{2}$	0.00
Stratospheric ozone depletion	kg CFC11-eq	$8.08 \cdot 10^{-5}$	$2.20 \cdot 10^{-5}$	$5.87 \cdot 10^{-5}$	0.00
Ionizing radiation	kBq Co-60-eq	$1.94 \cdot 10^{1}$	$3.66 \cdot 10^{0}$	$1.57 \cdot 10^{1}$	0.00
Ozone formation, Human health	kg NOx-eq	$5.28 \cdot 10^{-1}$	$1.16 \cdot 10^{-1}$	$4.12 \cdot 10^{-1}$	0.00
Fine particulate matter formation	kg PM2.5-eq	$4.63 \cdot 10^{-1}$	$1.08 \cdot 10^{-1}$	$3.55 \cdot 10^{-1}$	0.00
Ozone formation, Terrestrial ecosystems	kg NOx-eq	$5.37 \cdot 10^{-1}$	$1.20 \cdot 10^{-1}$	$4.17 \cdot 10^{-1}$	0.00
Terrestrial acidification	kg SO_2-eq	$9.28 \cdot 10^{-1}$	$2.51 \cdot 10^{-1}$	$6.77 \cdot 10^{-1}$	0.00
Freshwater eutrophication	kg P-eq	$1.27 \cdot 10^{-1}$	$4.60 \cdot 10^{-2}$	$8.13 \cdot 10^{-2}$	0.00
Marine eutrophication	kg N-eq	$1.06 \cdot 10^{-2}$	$4.95 \cdot 10^{-3}$	$5.67 \cdot 10^{-3}$	0.00
Terrestrial ecotoxicity	kg 1,4-DCB	$8.89 \cdot 10^{2}$	$7.48 \cdot 10^{2}$	$1.42 \cdot 10^{2}$	0.00
Freshwater ecotoxicity	kg 1,4-DCB	$4.38 \cdot 10^{1}$	$3.93 \cdot 10^{1}$	$4.58 \cdot 10^{0}$	0.00
Marine ecotoxicity	kg 1,4-DCB	$5.46 \cdot 10^{1}$	$4.84 \cdot 10^{1}$	$6.27 \cdot 10^{0}$	0.00
Human carcinogenic toxicity	kg 1,4-DCB	$1.26 \cdot 10^{1}$	$6.83 \cdot 10^{0}$	$5.77 \cdot 10^{0}$	0.00
Human non-carcinogenic toxicity	kg 1,4-DCB	$4.55 \cdot 10^{2}$	$2.28 \cdot 10^{2}$	$2.26 \cdot 10^{2}$	0.00
Land use	m^2a crop-eq	$1.38 \cdot 10^{2}$	$1.45 \cdot 10^{0}$	$1.36 \cdot 10^{2}$	0.00
Mineral resource scarcity	kg Cu-eq	$9.18 \cdot 10^{-1}$	$8.23 \cdot 10^{-1}$	$9.51 \cdot 10^{-2}$	0.00
Fossil resource scarcity	kg oil-eq	$5.79 \cdot 10^{1}$	$1.58 \cdot 10^{1}$	$4.21 \cdot 10^{1}$	0.00
Water consumption	m^3	$5.12 \cdot 10^{0}$	$2.10 \cdot 10^{0}$	$3.02 \cdot 10^{0}$	0.00

Table S20. ReCiPe 2016 v1.03 midpoint (H) results for the Boudouard scenario within the CDR expanded system (i.e., B_CDR).

Impact category	Unit	Total	FT electrofuels	Biomass gasification	Carbon dioxide removal
Global warming	kg CO_2-eq	$-2.72\cdot10^{2}$	$3.17\cdot10^{0}$	$-2.41\cdot10^{2}$	$-3.48\cdot10^{1}$
Stratospheric ozone depletion	kg CFC11-eq	$7.80\cdot10^{-5}$	$1.24\cdot10^{-5}$	$5.87\cdot10^{-5}$	$6.95\cdot10^{-6}$
Ionizing radiation	kBq Co-60-eq	$1.91\cdot10^{1}$	$1.70\cdot10^{0}$	$1.57\cdot10^{1}$	$1.64\cdot10^{0}$
Ozone formation, Human health	kg NOx-eq	$5.13\cdot10^{-1}$	$6.35\cdot10^{-2}$	$4.12\cdot10^{-1}$	$3.80\cdot10^{-2}$
Fine particulate matter formation	kg PM2.5-eq	$4.45\cdot10^{-1}$	$5.93\cdot10^{-2}$	$3.55\cdot10^{-1}$	$3.12\cdot10^{-2}$
Ozone formation, Terrestrial ecosystems	kg NOx-eq	$5.21\cdot10^{-1}$	$6.55\cdot10^{-2}$	$4.17\cdot10^{-1}$	$3.89\cdot10^{-2}$
Terrestrial acidification	kg SO_2-eq	$8.88\cdot10^{-1}$	$1.41\cdot10^{-1}$	$6.77\cdot10^{-1}$	$6.95\cdot10^{-2}$
Freshwater eutrophication	kg P-eq	$1.16\cdot10^{-1}$	$2.75\cdot10^{-2}$	$8.13\cdot10^{-2}$	$7.08\cdot10^{-3}$
Marine eutrophication	kg N-eq	$9.27\cdot10^{-3}$	$3.09\cdot10^{-3}$	$5.67\cdot10^{-3}$	$5.09\cdot10^{-4}$
Terrestrial ecotoxicity	kg 1,4-DCB	$6.51\cdot10^{2}$	$4.91\cdot10^{2}$	$1.42\cdot10^{2}$	$1.91\cdot10^{1}$
Freshwater ecotoxicity	kg 1,4-DCB	$3.09\cdot10^{1}$	$2.60\cdot10^{1}$	$4.58\cdot10^{0}$	$3.43\cdot10^{-1}$
Marine ecotoxicity	kg 1,4-DCB	$3.88\cdot10^{1}$	$3.20\cdot10^{1}$	$6.27\cdot10^{0}$	$4.79\cdot10^{-1}$
Human carcinogenic toxicity	kg 1,4-DCB	$1.05\cdot10^{1}$	$4.18\cdot10^{0}$	$5.77\cdot10^{0}$	$5.50\cdot10^{-1}$
Human non-carcinogenic toxicity	kg 1,4-DCB	$3.84\cdot10^{2}$	$1.48\cdot10^{2}$	$2.26\cdot10^{2}$	$9.29\cdot10^{0}$
Land use	m^2a crop-eq	$1.37\cdot10^{2}$	$8.35\cdot10^{-1}$	$1.36\cdot10^{2}$	$3.34\cdot10^{-1}$
Mineral resource scarcity	kg Cu-eq	$6.49\cdot10^{-1}$	$5.38\cdot10^{-1}$	$9.51\cdot10^{-2}$	$1.58\cdot10^{-2}$
Fossil resource scarcity	kg oil-eq	$5.99\cdot10^{1}$	$8.01\cdot10^{0}$	$4.21\cdot10^{1}$	$9.84\cdot10^{0}$
Water consumption	m^3	$3.75\cdot10^{0}$	$3.81\cdot10^{-1}$	$3.02\cdot10^{0}$	$3.48\cdot10^{-1}$

D.2. Endpoint results

Table S21. ReCiPe 2016 v1.03 endpoint (H) results for the RWGS scenario within the IH expanded system (i.e., RWGS_IH).

Damage category	Unit	Total	FT electrofuels	Biomass gasification	High temperature heating
Global warming	DALY	$2.55 \cdot 10^{-4}$	$1.41 \cdot 10^{-4}$	$7.71 \cdot 10^{-5}$	$3.61 \cdot 10^{-5}$
Stratospheric ozone depletion	species·yr	$1.10 \cdot 10^{-6}$	$1.62 \cdot 10^{-7}$	$8.27 \cdot 10^{-7}$	$1.09 \cdot 10^{-7}$
Water consumption	USD2013	$1.31 \cdot 10^{1}$	$4.47 \cdot 10^{0}$	$8.35 \cdot 10^{0}$	0.00

Table S22. ReCiPe 2016 v1.03 endpoint (H) results for the Boudouard scenario within the IH expanded system (i.e., B_IH).

Damage category	Unit	Total	FT electrofuels	Biomass gasification	High temperature heating
Global warming	DALY	$2.10 \cdot 10^{-4}$	$8.87 \cdot 10^{-5}$	$7.71 \cdot 10^{-5}$	$4.43 \cdot 10^{-5}$
Stratospheric ozone depletion	species·yr	$9.93 \cdot 10^{-7}$	$1.05 \cdot 10^{-7}$	$8.27 \cdot 10^{-7}$	$6.04 \cdot 10^{-8}$
Water consumption	USD2013	$1.14 \cdot 10^{1}$	$2.50 \cdot 10^{0}$	$8.35 \cdot 10^{0}$	$5.61 \cdot 10^{-1}$

Table S23. ReCiPe 2016 v1.03 endpoint (H) results for the RWGS scenario within the CDR expanded system (i.e., RWGS_CDR).

Damage category	Unit	Total	FT electrofuels	Biomass gasification	Carbon dioxide removal
Global warming	DALY	$2.18 \cdot 10^{-4}$	$1.41 \cdot 10^{-4}$	$7.71 \cdot 10^{-5}$	0.00
Stratospheric ozone depletion	species·yr	$9.90 \cdot 10^{-7}$	$1.62 \cdot 10^{-7}$	$8.27 \cdot 10^{-7}$	0.00
Water consumption	USD2013	$1.31 \cdot 10^{1}$	$4.47 \cdot 10^{0}$	$8.35 \cdot 10^{0}$	0.00

Table S24. ReCiPe 2016 v1.03 endpoint (H) results for the Boudouard scenario within the CDR expanded system (i.e., B_CDR).

Damage category	Unit	Total	FT electrofuels	Biomass gasification	Carbon dioxide removal
Global warming	DALY	$1.58 \cdot 10^{-4}$	$8.87 \cdot 10^{-5}$	$7.71 \cdot 10^{-5}$	$-7.99 \cdot 10^{-5}$
Stratospheric ozone depletion	species·yr	$8.67 \cdot 10^{-7}$	$1.05 \cdot 10^{-7}$	$8.27 \cdot 10^{-7}$	$-6.51 \cdot 10^{-8}$
Water consumption	USD2013	$1.38 \cdot 10^{1}$	$2.50 \cdot 10^{0}$	$8.35 \cdot 10^{0}$	$2.97 \cdot 10^{0}$

E. Economic assessment results

In this section, we show the economic contributions of each process unit per process, and the final cost breakdown per scenario.

Table S25. CAPEX contributions of the RWGS configuration updated to 2023.

Unit	Purchase cost [M$]	CAPEX [M$]
Compressor (centrifugal)	$2.10 \cdot 10^{1}$	$1.43 \cdot 10^{2}$
Heat exchanger (U-tube shell and tube)	$3.07 \cdot 10^{1}$	$2.09 \cdot 10^{2}$
Pump (single stage centrifugal)	$3.29 \cdot 10^{-2}$	$2.24 \cdot 10^{-1}$
Furnace (cylindrical)	$1.37 \cdot 10^{1}$	$9.35 \cdot 10^{1}$
Flash unit (pressure vessel)	$2.83 \cdot 10^{0}$	$1.93 \cdot 10^{1}$
Distillation column (pressure vessel+trays)	$6.47 \cdot 10^{-1}$	$4.41 \cdot 10^{0}$
RWGS reactor	$1.01 \cdot 10^{1}$	$6.88 \cdot 10^{1}$
FT reactor	$8.26 \cdot 10^{0}$	$5.62 \cdot 10^{1}$
HC reactor	$2.23 \cdot 10^{2}$	$1.52 \cdot 10^{3}$
PSA unit	$7.67 \cdot 10^{0}$	$1.25 \cdot 10^{1}$

Table S26. CAPEX contributions of the Boudouard configuration updated to 2023.

Unit	Purchase cost [M$]	CAPEX [M$]
Compressor (centrifugal)	$1.56 \cdot 10^{1}$	$1.08 \cdot 10^{2}$
Heat exchanger (U-tube shell and tube)	$1.71 \cdot 10^{1}$	$1.19 \cdot 10^{2}$
Pump (single stage centrifugal)	$3.42 \cdot 10^{-2}$	$2.38 \cdot 10^{-1}$
Furnace (cylindrical)	$1.30 \cdot 10^{1}$	$9.06 \cdot 10^{1}$
Flash unit (pressure vessel)	$6.80 \cdot 10^{-1}$	$4.73 \cdot 10^{0}$
Distillation column (pressure vessel+trays)	$6.47 \cdot 10^{-1}$	$4.50 \cdot 10^{0}$
Boudouard reactor	$9.80 \cdot 10^{0}$	$6.81 \cdot 10^{1}$
FT reactor	$7.92 \cdot 10^{0}$	$5.51 \cdot 10^{1}$
HC reactor	$2.33 \cdot 10^{2}$	$1.62 \cdot 10^{3}$
PSA unit	$7.94 \cdot 10^{0}$	$1.26 \cdot 10^{1}$

Table S27. CAPEX contributions of the biomass gasification process updated to 2023.

Unit	Purchase cost [M$]	CAPEX [M$]
Compressor (centrifugal)	$1.96 \cdot 10^{1}$	$1.36 \cdot 10^{2}$
Heat exchanger (U-tube shell and tube)	$5.35 \cdot 10^{0}$	$3.72 \cdot 10^{1}$
Flash unit (pressure vessel)	$3.79 \cdot 10^{0}$	$2.64 \cdot 10^{1}$
Gasifier	$3.39 \cdot 10^{1}$	$5.37 \cdot 10^{1}$
Water-gas shift (WGS) reactor	$9.96 \cdot 10^{-1}$	$1.58 \cdot 10^{0}$
PSA unit	$8.76 \cdot 10^{0}$	$1.39 \cdot 10^{1}$

Table S28. CAPEX contributions of the industrial heating process updated to 2023.

Unit	Purchase cost [M$]	CAPEX [M$]
Furnace (cylindrical)	$5.77 \cdot 10^{0}$	$3.93 \cdot 10^{1}$

Table S29. CAPEX contributions of the DACCS process updated to 2023.

Unit	Purchase cost [M$]	CAPEX [M$]
Compressor (centrifugal)	$6.18 \cdot 10^{0}$	$4.21 \cdot 10^{1}$
Heat exchanger (U-tube shell and tube)	$1.93 \cdot 10^{-1}$	$1.31 \cdot 10^{0}$

Table S30. Cost breakdown for the RWGS scenario within the IH expanded system (i.e., RWGS_IH).

**Contribution**	**Cost [$ / (functional unit)]**
H_2 (from wind electricity)	$7.65 \cdot 10^{1}$
CO_2 (from direct air capture)	$2.39 \cdot 10^{1}$
Heating (from natural gas)	0.00
Cooling (water from 20 to 25 ºC)	$1.38 \cdot 10^{0}$
Electricity (global mix)	$6.30 \cdot 10^{-1}$
Wastewater	$5.20 \cdot 10^{-1}$
Fixed OPEX	$2.07 \cdot 10^{0}$
CAPEX	$8.34 \cdot 10^{0}$
Biomass gasification	$5.8 \cdot 10^{1}$
Industrial heating	$2.70 \cdot 10^{-1}$

Table S31. Cost breakdown for the Boudouard scenario within the IH expanded system (i.e., B_IH).

**Contribution**	**Cost [$ / (functional unit)]**
H_2 (from wind electricity)	$5.09 \cdot 10^{1}$
CO_2 (from direct air capture)	$9.71 \cdot 10^{0}$
Heating (from natural gas)	$8.00 \cdot 10^{-2}$
Cooling (water from 20 to 25 ºC)	$1.50 \cdot 10^{-1}$
Electricity (global mix)	$3.30 \cdot 10^{-1}$
Wastewater	$3.50 \cdot 10^{-1}$
Fixed OPEX	$1.97 \cdot 10^{0}$
CAPEX	$8.15 \cdot 10^{0}$
Biomass gasification	$5.8 \cdot 10^{1}$
Industrial heating	$2.56 \cdot 10^{1}$

Table S32. Cost breakdown for the RWGS scenario within the CDR expanded system (i.e., RWGS_CDR).

Contribution	Cost [$ / (functional unit)]
H_2 (from wind electricity)	$7.65 \cdot 10^{1}$
CO_2 (from direct air capture)	$2.39 \cdot 10^{1}$
Heating (from natural gas)	0.00
Cooling (water from 20 to 25 ºC)	$1.38 \cdot 10^{0}$
Electricity (global mix)	$6.30 \cdot 10^{-1}$
Wastewater	$5.20 \cdot 10^{-1}$
Fixed OPEX	$2.07 \cdot 10^{0}$
CAPEX	$8.34 \cdot 10^{0}$
Biomass gasification	$5.8 \cdot 10^{1}$
CDR	0.00

Table S33. Cost breakdown for the Boudouard scenario within the CDR expanded system (i.e., B_CDR).

Contribution	Cost [$ / (functional unit)]
H_2 (from wind electricity)	$5.09 \cdot 10^{1}$
CO_2 (from direct air capture)	$9.71 \cdot 10^{0}$
Heating (from natural gas)	$8.00 \cdot 10^{-2}$
Cooling (water from 20 to 25 ºC)	$1.50 \cdot 10^{-1}$
Electricity (global mix)	$3.30 \cdot 10^{-1}$
Wastewater	$3.50 \cdot 10^{-1}$
Fixed OPEX	$1.97 \cdot 10^{0}$
CAPEX	$8.15 \cdot 10^{0}$
Biomass gasification	$5.8 \cdot 10^{1}$
CDR	$3.20 \cdot 10^{1}$

References

(1) Medrano-García, J. D.; Chagas, M. T.; Guillén-Gosálbez, G. Integrating the Reverse Boudouard Reaction for a More Efficient Green Methanol Synthesis from CO_2 and Renewable Energy. *ACS Sustainable Chem. Eng.* **2025**, *13* (19), 7088–7097. https://doi.org/10.1021/acssuschemeng.5c01021.

(2) Intergovernmental Panel on Climate Change (IPCC). *2019 Refinement to the 2006 IPCC Guidelines for National Greenhouse Gas Inventories*; 2019. https://www.ipcc-nggip.iges.or.jp/public/2019rf/pdf/4_Volume4/19R_V4_Ch02_Ap4_Biochar.pdf (accessed 2025-02-03).

(3) Keith, D. W.; Holmes, G.; St. Angelo, D.; Heidel, K. A Process for Capturing CO2 from the Atmosphere. *Joule* **2018**, *2* (8), 1573–1594. https://doi.org/10.1016/j.joule.2018.05.006.

(4) Medrano-García, J. D.; Calvo-Serrano, R.; Tian, H.; Guillén-Gosálbez, G. Win–Win More Sustainable Routes for Acetic Acid Synthesis. *ACS Sustainable Chem. Eng.* **2025**, *13* (4), 1522–1531. https://doi.org/10.1021/acssuschemeng.4c07324.

(5) Medrano-García, J. D.; Giulimondi, V.; Ceruti, A.; Zichittella, G.; Pérez-Ramírez, J.; Guillén-Gosálbez, G. Economic and Environmental Competitiveness of Ethane-Based Technologies for Vinyl Chloride Synthesis. *ACS Sustainable Chem. Eng.* **2023**, *11* (35), 13062–13069. https://doi.org/10.1021/acssuschemeng.3c03006.

(6) Medrano-García, J. D.; Charalambous, M. A.; Guillén-Gosálbez, G. Economic and Environmental Barriers of CO_2-Based Fischer-Tropsch Electro-Diesel. *ACS Sustainable Chem. Eng.* **2022**, *10* (36), 11751–11759. https://doi.org/10.1021/acssuschemeng.2c01983.

(7) Nabera, A.; Martín, A. J.; Istrate, R.; Pérez-Ramírez, J.; Guillén-Gosálbez, G. Integrating Climate Policies in the Sustainability Analysis of Green Chemicals. *Green Chem.* **2024**, *26* (11), 6461–6469. https://doi.org/10.1039/D4GC00392F.

(8) Salah, C.; Istrate, R.; Bjørn, A.; Tulus, V.; Pérez-Ramírez, J.; Guillén-Gosálbez, G. Environmental Benefits of Circular Ethylene Production from Polymer Waste. *ACS Sustainable Chem. Eng.* **2024**, *12* (37), 13897–13906. https://doi.org/10.1021/acssuschemeng.4c04241.

(9) Sinnott, R.; Towler, G. Costing and Project Evaluation. In *Chemical Engineering Design*; Elsevier, 2020; pp 275–369. https://doi.org/10.1016/B978-0-08-102599-4.00006-0.

(10) König, D. H.; Freiberg, M.; Dietrich, R.-U.; Wörner, A. Techno-Economic Study of the Storage of Fluctuating Renewable Energy in Liquid Hydrocarbons. *Fuel* **2015**, *159*, 289–297. https://doi.org/10.1016/j.fuel.2015.06.085.

(11) Onel, O.; Niziolek, A. M.; Elia, J. A.; Baliban, R. C.; Floudas, C. A. Biomass and Natural Gas to Liquid Transportation Fuels and Olefins (BGTL+C2_C4): Process Synthesis and Global Optimization. *Ind. Eng. Chem. Res.* **2015**, *54* (1), 359–385. https://doi.org/10.1021/ie503979b.
